# Supplementary material for: Informing climate-health adaptation options through mapping the needs and potential for integrated climate-driven early warning forecasting systems in South Asia—A scoping review
Source: PLoS One. 2024 Oct 24;19(10):e0309757. doi: 10.1371/journal.pone.0309757 (PMC11500899; doi:10.1371/journal.pone.0309757)
Supplement: S1 Table — (DOCX) [file pone.0309757.s002.docx]

**S1 Table. Summary characteristics of reviewed studies**

| **Author(s)** | **Year** | **DOI** | **Document type** | **Article Title:** | **Main question/ purpose for the study** | **Study design** | **Country** |
| --- | --- | --- | --- | --- | --- | --- | --- |
| Rahman, Md Mostafizur; Bodrud-Doza, Md; Shammi, Mashura; Islam, Abu Reza Md Towfiqul; Khan, Abu Sadat Moniruzzaman | 2021 | 10.1016/j.envres.2020.110303 | Article | COVID-19 pandemic, dengue epidemic, and climate change vulnerability in Bangladesh: Scenario assessment for strategic management and policy implications | This study aimed to assess the potential impact of a simultaneous strike of climatic hazards and infectious disease outbreaks and their possible strategic management in Bangladesh under different scenarios | Mixed methods | Bangladesh |
| Anoopkumar AN, Aneesh EM | 2021 | 10.1007/s10668-021-01792-4 | Article | A critical assessment of mosquito control and the influence of climate change on mosquito-borne disease epidemics | This review offers invaluable insights into severe mosquito-borne diseases, various vector control strategies, and the influence of climate change in mosquito-borne disease transmission. | Quantitative | India |
| Khan, AM; Dutta, P; Khan, SA; Mahanta, J | 2003 |  | Article | A focus of lymphatic filariasis in a tea garden worker community of central Assam | Aim to ascertain the current status of filariasis by 2020 globally | Quantitative | India |
| Rajvanshi, H; Bharti, PK; Nisar, S; Jayswar, H; Mishra, AK; Sharma, RK; Saha, KB; Shukla, MM; Wattal, SL; Das, A; Kaur, H; Anvikar, AR; Khan, A; Kshirsagar, N; Dash, AP; Lal, AA | 2021 | 10.1186/s12936-021-03607-3 | Article | A model for malaria elimination based on learnings from the Malaria Elimination Demonstration Project, Mandla district, Madhya Pradesh | To demonstrate that malaria can be eliminated from a high malaria endemic district and to develop a model for malaria elimination using the lessons learned and knowledge acquired from the demonstration project. | Quantitative | India |
| Pathak VK, Mohan M | 2019 | 10.4103/jfmpc.jfmpc_716_19 | Review | A notorious vector-borne disease: Dengue fever, its evolution as public health threat. | To review the origin, history, and current epidemiology of dengue, its transmission, factors associated and the treatment options available | Quantitative | India |
| Dutta SOMENATH, Balasubramaniam R, Jagtap MAHENDRA, Awate PRADEEP, Kulkarni NAHUSH, Danish MD, Deshpande S, Satpute U, Wayal R, Bhagbat P, Nambier BINDU, Kulkarni D, Bile L, Kamble PV, Ghosh KRIPAN, Sawaisarje GK, Khedikar SIRISH, Patil CHETNA, Alam OSAID, Sahai AK | 2021 | 10.54302/mausam.v72i2.611 | Article | A pilot study on assessing the effect of climate on the incidence of vector borne disease at Pune and Pimpri-Chinchwad area, Maharashtra | To understand the role of different climatic parameters in the locally outbreak of VBD like Malaria and Dengue | Qualitative | India |
| Ramaiah KD, Das PK, Appavoo NC, Ramu K, Augustin DJ, Kumar KN, Chandrakala AV | 2000 | 10.1046/j.1365-3156.2000.00659.x | Article | A programme to eliminate lymphatic filariasis in Tamil Nadu state, India: compliance with annual single-dose DEC mass treatment and some related operational aspects. | Reports on DEC distribution and compliance with treatment in a large-scale annual single-dose mass treatment programme to eliminate lymphatic filariasis in the south Indian state of Tamil Nadu | Quantitative | India |
| Liyanage P, Tissera H, Sewe M, Quam M, Amarasinghe A, Palihawadana P, Wilder-Smith A, Louis VR, Tozan Y, Rocklöv J | 2016 | 10.3390/ijerph13111087 | Article | A Spatial Hierarchical Analysis of the Temporal Influences of the El Nino-Southern Oscillation and Weather on Dengue in Kalutara District, Sri Lanka | This study focuses on quantifying the influence of weather variability on dengue incidence over 10 Medical Officer of Health (MOH) divisions of Kalutara district. | Quantitative | Sri Lanka |
| Ehelepola ND, Ariyaratne K, Buddhadasa WM, Ratnayake S, Wickramasinghe M | 2015 | 10.1186/s40249-015-0075-8 | Article | A study of the correlation between dengue and weather in Kandy City, Sri Lanka (2003-2012) and lessons learned | This study aimed to identify a dengue weather correlation pattern in Kandy, Sri Lanka, compare the results with results of similar studies, and establish ways for better control and prevention of dengue. | Mixed methods | Sri Lanka |
| Rahman MA, Yahathugoda TC, Tojo B, Premaratne P, Nagaoka F, Takagi H, Kannathasan S, Murugananthan A, Weerasooriya MV, Itoh M | 2019 | 10.1016/j.parint.2018.10.003 | Article | A surveillance system for lymphatic filariasis after its elimination in Sri Lanka. | Investigate LF transmission in Trincomalee district where a surveillance program was not carried out | Mixed methods | Sri Lanka |
| Regmi K, Kunwar A, Ortega L | 2016 | 10.3402/iee.v6.30822 | Review | A systematic review of knowledge, attitudes and beliefs about malaria among the South Asian population | To understand the knowledge, attitudes and beliefs about malaria in South Asian communities. | Quantitative | South Asia |
| Khan E, Barr KL, Farooqi JQ, Prakoso D, Abbas A, Khan ZY, Ashi S, Imtiaz K, Aziz Z, Malik F, Lednicky JA, Long MT | 2018 | 10.3389/fpubh.2018.00020 | Article | Added to library on 10 Feb 2022 by You Human West Nile Virus Disease Outbreak in Pakistan, 2015-2016. | To describe the active and persistent circulation of WNV in humans in the southern region of Pakistan | Mixed methods | Pakistan |
| Babu BV, Rath K, Kerketta AS, Swain BK, Mishra S, Kar SK | 2006 | 10.1016/j.trstmh.2005.07.016, | Article | Adverse reactions following mass drug administration during the Programme to Eliminate Lymphatic Filariasis in Orissa State, India | This paper reports the frequency and types of adverse reactions during two MDAs during January 2002 and September 2004 in the State of Orissa, India. | Quantitative | India |
| Kumar A, Hosmani R, Jadhav S, de Sousa T, Mohanty A, Naik M, Shettigar A, Kale S, Valecha N, Chery L, Rathod PK | 2016 | 10.1186/s12936-016-1177-x | Article | Anopheles subpictus carry human malaria parasites in an urban area of Western India and may facilitate perennial malaria transmission | Analyses the human and bovine blood meal and the presence of human Plasmodia | Quantitative | India |
| SAPANA ASHOK SASANE, A. S. JADHAV, RABINDRA K. BARIK, G. KRISHNAKUMAR and V. RAGHAVSWAMY | 2019 | 10.54302/mausam.v70i4.264 | Article | Application of spatial technology in malaria information infrastructure mapping with climate change perspective in Maharashtra, India | The present paper attempts to study the patterns and trends of the incidence of Malaria in urban landscapes of Maharashtra by analyzing the data of fourteen years from 2001 to 2014. | Qualitative | India |
| Chandy S, Ramanathan K, Manoharan A, Mathai D, Baruah K | 2013 | 10.4103/0255-0857.115640 | Article | Assessing effect of climate on the incidence of dengue in Tamil Nadu | To assess the association of local climate with dengue incidence, in two geographically distinct districts in Tamil Nadu | Mixed methods | India |
| Singh N, Mall RK, Banerjee T, Gupta A | 2021 | 10.1016/j.scitotenv.2021.148769 | Article | Association between climate and infectious diseases among children in Varanasi city, India: A prospective cohort study. | The urgent need to test the hypothesis of whether there exists an indirect and direct cause-and-effect relationship between climate variability and paediatric health | Quantitative | India |
| Borah J, Dutta P, Khan SA, Mahanta J | 2013 | 10.1007/s10393-013-0849-z | Article | Association of weather and anthropogenic factors for transmission of Japanese encephalitis in an endemic area of India | Aims to determine the relationship between meteorological and anthropogenic factors and JE occurrence in vulnerable areas and to provide early warning signs to the local health or administrative authorities for management in these areas through community participation | Quantitative | India |
| Prusty D, Gupta N, Upadhyay A, Dar A, Naik B, Kumar N, Prajapati VK | 2021 | 10.1016/j.meegid.2021.104987 | Review paper | Asymptomatic malaria infection prevailing risks for human health and malaria elimination | Highlight the impacts of asymptomatic malaria on human health and its contribution to disease transmission | Quantitative | India |
| Paramanik M, Sarkar N, Chandra G | 2020 | 10.4103/0972-9062.313970 | Article | Awareness and impact of Lymphatic Filariasis among school children in rural endemic areas of West Bengal, India | This study assessed the filarial epidemiology as well as awareness about the disease among school children in the rural areas of Bankura district, West Bengal, India. | Quantitative | India |
| Nilgiriwala KS | 2018 | 10.4103/ijph.IJPH_306_17 | Review | Basis of Science Policies for Infectious Disease Challenges in India | Gives an insight into the strengths and limitations of the basis of some of the contemporary science policies in India that are drafted and implemented to combat the challenges of infectious diseases. | Quantitative | India |
| Sharma VP | 2012 | PMC3612321 | Review | Battling malaria iceberg incorporating strategic reforms in achieving Millennium Development Goals & malaria elimination in India | Outline the historical account of malaria and methods of control that have lifted the national economy in many countries | Mixed methods | India |
| Zaman K, Ryun Kim D, Ali M, Ahmmed F, Im J, Islam MT, Khan AI, Yunus M, Khan MA, Marks F, Qadri F, Kim J, Clemens JD | 2020 | 10.1016/j.ijid.2020.02.055 | Article | Can cholera 'hotspots' be converted to cholera 'coldspots' in cholera endemic countries? The Matlab, Bangladesh experience | To analyze the trend in cholera incidence during the years 1974–2018 in Matlab, Bangladesh | Quantitative | Bangladesh |
| Malavige GN, Jeewandara C, Ghouse A, Somathilake G, Tissera H | 2021 | 10.1371/journal.pntd.0009624 | Article | Changing epidemiology of dengue in Sri Lanka-Challenges for the future | discuss the changes in dengue epidemiology in Sri Lanka in relation to changes in age distribution, changes in seroprevalence rates over time, and possible reasons contributing to such changes. | Qualitative | Sri Lanka |
| Rai SK | 2018 | 10.1007/978-981-10-7572-8_3 | Article | Changing trend of infectious diseases in nepal | To address the present situation of disease infestation through multi-sectoral plan and strategies | Quantitative | Nepal |
| Dharmawardena P, Premaratne RG, Gunasekera WM, Hewawitarane M, Mendis K, Fernando D | 2015 | 10.1186/s12936-015-0697-0 | Review | Characterization of imported malaria, the largest threat to sustained malaria elimination from Sri Lanka | This study reports on two clusters of imported malaria, one amongst Pakistani asylum-seekers resident in a malaria non-endemic area in Sri Lanka and the other amongst local fishermen who returned from Sierra Leone | Qualitative | Sri Lanka |
| Chowdhury P, Khan SA, Dutta P, Topno R, Mahanta J | 2014 | 10.1016/j.cimid.2013.10.006 | Article | Characterization of West Nile virus (WNV) isolates from Assam, India: Insights into the circulating WNV in northeastern India | To establish and characterize the currently circulating strain/s of WNV in the NER of India. | Quantitative | India |
| Reid HL, Haque U, Roy S, Islam N, Clements AC | 2012 | 10.1186/1475-2875-11-170 | Article | Characterizing the spatial and temporal variation of malaria incidence in Bangladesh, 2007 | To understand the spatial and temporal patterns in malaria risk and the determinants driving the variation | Quantitative | Bangladesh |
| Debes AK, Ali M, Azman AS, Yunus M, Sack DA | 2016 | 10.1093/ije/dyw267 | Article | Cholera cases cluster in time and space in Matlab, Bangladesh: implications for targeted preventive interventions. | To estimate the spatial and temporal extent of the zone of increased risk around cholera cases. | Quantitative | Bangladesh |
| Pascual M, Rodó X, Ellner SP, Colwell R, Bouma MJ | 2000 | 10.1126/science.289.5485.1766 | Article | Cholera dynamics and El Nino-Southern Oscillation | Examine the associations between cholera and ENSO and between cholera and climate at interannual time scales, using an 18-year record from Bangladesh where the disease is endemic | Quantitative | Bangladesh |
| Siddique AK, Zaman K, Baqui AH, Akram K, Mutsuddy P, Eusof A, Haider K, Islam S, Sack RB | 1992 | PMID: 1500643 | Article | CHOLERA EPIDEMICS IN BANGLADESH - 1985-1991 | Determine the magnitude of the epidemic disease and some of the related public health issues in rural Bangladesh | Qualitative | Bangaldesh |
| Martinez PP, Reiner RC, Cash BA, Rodó X, Shahjahan Mondal M, Roy M, Yunus M, Faruque AS, Huq S, King AA, Pascual M | 2017 | 10.1371/journal.pone.0172355 | Article | Cholera forecast for Dhaka, Bangladesh, with the 2015-2016 El Niño: Lessons learned. | To evaluate the predictability of cholera dynamics for the city in recent times based on two transmission models | Quantitative | Bangladesh |
| Lopez AL, Dutta S, Qadri F, Sovann L, Pandey BD, Bin Hamzah WM, Memon I, Iamsirithaworn S, Dang DA, Chowdhury F, Heng S, Kanungo S, Mogasale V, Sultan A, Ylade M | 2020 | 10.1016/j.vaccine.2019.07.035, | Review paper | Cholera in selected countries in Asia. | To provide a sub-regional assessment of cholera cases in Asia | Quantitative | Bangladesh, Cambodia, India, Malaysia, Nepal, Pakistan, Philippines, Thailand and Vietnam |
| Rahman MS, Karamehic-Muratovic A, Baghbanzadeh M, Amrin M, Zafar S, Rahman NN, Shirina SU, Haque U | 2021 | 10.1093/trstmh/traa093 | Article | Climate change and dengue fever knowledge, attitudes and practices in Bangladesh: a social media-based cross-sectional survey | This study investigated the knowledge, attitudes and practices (KAP) among university students in Bangladesh and significant factors associated with their prevention practices related to climate change and DF. | Quantitative | Bangladesh |
| Bhandari D, Bi P, Sherchand JB, Dhimal M, Hanson-Easey S | 2020 | 10.1016/j.actatropica.2020.105337 | Review | Climate change and infectious disease research in Nepal: Are the available prerequisites supportive enough to researchers? | identify and characterise basic requirements that are hindering the progress of climate change and infectious disease research in Nepal | Quantitative | Nepal |
| Anwar A, Anwar S, Ayub M, Nawaz F, Hyder S, Khan N, Malik I | 2019 | PMID: 31993386 | Article | Climate Change and Infectious Diseases: Evidence from Highly Vulnerable Countries | This study was conducted to examine the effect of climate change and some socio-economic factors on incidence of infectious diseases. | Qualitative | India; Bangladesh |
| Dhimal M, Kramer IM, Phuyal P, Budhathoki SS, Hartke J, Ahrens B, Kuch U, Groneberg DA, Nepal S, Liu QY, Huang CR, CissÉ G, Ebi KL, KlingelhÖfer D, Müller R | 2021 | 10.1016/j.accre.2021.05.003 | Review | Climate change and its association with the expansion of vectors and vector-borne diseases in the Hindu Kush Himalayan region: A systematic synthesis of the literature | To identify the most significant variables to be considered for evidence-based trend estimates of the effects of climate change on VBDs and their vectors in the HKH region. | Qualitative | Hindu Kush Himalayan (HKH) region |
| Karmakar M, Pradhan MM | 2019 | 10.1007/s11069-019-03594-4 | Article | Climate change and public health: a study of vector-borne diseases in Odisha, India | The study focuses on climate change impacts in terms of increased severity, frequency and spread of vector-borne diseases. | Quantitative | India |
| Singh BB, Sharma R, Gill JP, Aulakh RS, Banga HS | 2011 | 10.20506/rst.30.3.2073 | Article | Climate change and water resources of Himalayan region—review of impacts and implication | Discuss zoonotic diseases and their vectors and the effect of climate change on important zoonoses in India | Quantitative | India |
| Udayanga L, Gunathilaka N, Iqbal MCM, Abeyewickreme W | 2020 | 10.1186/s40249-020-00717-z | Article | Climate change induced vulnerability and adaption for dengue incidence in Colombo and Kandy districts: the detailed investigation in Sri Lanka. | To evaluate the climate change induced socio-economic vulnerability of local communities to dengue in Colombo and Kandy districts of Sri Lanka. | Quantitative | Sri Lanka |
| Ahmed, Toqeer; Zounemat-Kermani, Mohammad; Scholz, Miklas | 2020 | 10.3390/ijerph17228518 | Review | Climate Change, Water Quality and Water-Related Challenges: A Review with Focus on Pakistan | Analyze climate variability and water-related disasters as well as their impacts on human health. Finally, some key recommendations are made for policy-makers. | Quantitative | Pakistan |
| Baeza A, Bouma MJ, Dobson AP, Dhiman R, Srivastava HC, Pascual M | 2011 | 10.1186/1475-2875-10-190 | Article | Climate forcing and desert malaria: the effect of irrigation | focuses on the response of malaria epidemics to rainfall forcing and how this response is affected by increasing irrigation. | Quantitative | India |
| Servadio JL, Rosenthal SR, Carlson L, Bauer C | 2018 | 10.1016/j.jiph.2017.12.006 | Article | Climate patterns and mosquito-borne disease outbreaks in South and Southeast Asia | This study aims to make use of a large data set to determine how risk of mosquito-borne infectious disease outbreaks relates to the highest monthly average temperature and precipitation for each year in South and Southeast Asia. | Quantitative | South Asia |
| Islam S, Haque CE, Hossain S, Hanesiak J | 2021 | 10.3390/atmos12070905 | Article | Climate Variability, Dengue Vector Abundance and Dengue Fever Cases in Dhaka, Bangladesh: A Time-Series Study | Examine the effects of climatic factors on vector abundance and subsequent effects on dengue cases of Dhaka city, Bangladesh. | Quantitative | Bangladesh |
| Perez-Saez J, King AA, Rinaldo A, Yunus M, Faruque ASG, Pascual M | 2017 | 10.1016/j.advwatres.2016.11.013 | Article | Climate-driven endemic cholera is modulated by human mobility in a megacity | Build on an established probabilistic spatial model to investigate the importance and role of human mobility in modulating spatial cholera | Qualitative | Bangladesh |
| Hafiz I, Graves P, Haq R, Flora MS, Kelly-Hope LA | 2015 | 10.1093/trstmh/trv084 | Article | Clinical case estimates of lymphatic filariasis in an endemic district of Bangladesh after a decade of mass drug administration | This survey estimated the current number of clinical cases in a historically endemic district after a decade of mass drug administration (MDA). | Quantitative | Bangladesh |
| Lonappan S, Golecha R, Balakrish Nair G | 2020 | 10.1016/j.vaccine.2019.08.022 | Article | Contrasts, contradictions and control of cholera | discusses the contrasts and contradictions of cholera, its control and its unpredictable nature. | Qualitative | Asia and other parts of the world |
| Zahirul Islam M, Rutherford S, Phung D, Uzzaman MN, Baum S, Huda MM, Asaduzzaman M, Talukder MRR, Chu C | 2018 | 10.7759/cureus.3398 | Article | Correlates of Climate Variability and Dengue Fever in Two Metropolitan Cities in Bangladesh | Aims to assess the correlation of temperature, humidity, and rainfall on dengue fever in two major urban cities (Dhaka and Chittagong) in Bangladesh | Qualitative | Bangladesh |
| Babu BV, Babu GR | 2014 | 10.1093/trstmh/tru057 | Review | Coverage of, and compliance with, mass drug administration under the programme to eliminate lymphatic filariasis in India: a systematic review | To systematically review published studies on the coverage of and compliance with MDA under the PELF in India | Qualitative | India |
| Ki Beom Park,Hongray Howrelia Patnaik,Tae-Yun Kim,Yong Hun Jo,Nam-Yeon Kim,Sung-Chan Yang,Wook-Gyo Lee,Hee-Il Lee,Shin-Hyeong Cho,Yeon Soo Han | 2021 | 10.1111/1748-5967.12439 | Review | Current trends in large‐scale viral surveillance methods in mosquitoes | This study elaborates the geographic spread and pathogenicity of the viruses with a reservoir in the mosquito host and clinical manifestations in humans | Quantitative | India |
| Upadhyayula SM, Mutheneni SR, Kadiri MR, Kumaraswamy S, Nelaturu SC | 2012 | 10.1371/journal.pone.0039970 | Article | Data base management system for lymphatic filariasis--a neglected tropical disease. | Develop database on lymphatic filariasis | Qualitative | India |
| Khuntia HK, Ramamurthy T, Bal M, Pati S, Ranjit M | 2021 | 10.1017/S0950268821001266 | Article | Decades of cholera in Odisha, India (1993-2015): lessons learned and the ways forward | Provide comprehensive report on epidemiology of cholera in Odisha | Quantitative | India |
| Dev V, Manguin S | 2021 | 10.1016/j.actatropica.2021.106040 | Review | Defeating malaria in the North-East region: the forerunner for malaria elimination in India. | Discusses the actions to be taken to galvanize malaria control in the North-East region of India | Quantitative | India |
| Raheel U, Faheem M, Riaz MN, Kanwal N, Javed F, Zaidi NU, Qadri I | 2011 | 10.3855/jidc.1017 | Review | Dengue fever in the Indian Subcontinent: an overview | Discuss annual cases of DF/DHF in India, Pakistan, Bangladesh and Sri Lanka and possible factors involved in DF outbreaks. | Mixed methods | India |
| Bostan N, Javed S, Nabgha-E-Amen, Eqani SA, Tahir F, Bokhari H | 2017 | 10.1002/rmv.1899 | Review | Dengue fever virus in Pakistan: effects of seasonal pattern and temperature change on distribution of vector and virus. | Review the climatic as well as host- and vector-associated factors involved in the outbreak of dengue epidemics in Pakistan and highlight high-risk zones in the country | Quantitative | Pakistan |
| Kakarla SG, Bhimala KR, Kadiri MR, Kumaraswamy S, Mutheneni SR | 2020 | 10.1016/j.scitotenv.2020.140336 | Article | Dengue situation in India: Suitability and transmission potential model for present and projected climate change scenarios | To understand the expansion of dengue transmission potential into a new geographic region in the present and projected emission scenarios with high spatial and temporal resolution over India. | Quantitative | India |
| Jisamerin J, Mohamedkalifa A, Gaur A, Geetha J, Sakthivadivel V | 2021 | 10.7759/cureus.18500 | Article | Dengue: A neglected disease of concern | To analyze the demographic and clinical profile of dengue patients admitted to a tertiary care center in Tamilnadu | Quantitative | India |
| Mangeard-Lourme J, Robert de Arquer G, Parasa J, Singh RK, Satle N, Mamhidi R | 2020 | 10.47276/lr.91.4.367 | Article | Depression and anxiety in people affected by leprosy and lymphatic filariasis: a cross-sectional study in four States in India | The aim of the current study is to assess the scale for depression and anxiety among people affected by leprosy and LF seeking medical care in India, and the main risks associated with poor mental health. | Quantitative | India |
| Khan SA, Chowdhury P, Choudhury P, Dutta P | 2017 | 10.1186/s13071-016-1948-9 | Article | Detection of West Nile virus in six mosquito species in synchrony with seroconversion among sentinel chickens in India. | Identified local mosquito species for evidence of WNV infection along with seroconversion among sentinel chickens. | Quantitative | India |
| Zohaib, Ali; Niazi, Saifullah Khan; Saqib, Muhammad; Sajid, Muhammad Sohail; Khan, Iahtasham; Sial, Awais-ur-Rahman; Athar, Muhammad Ammar; Taj, Zeeshan; Abbas, Ghazanfar; Rathore, Muhammad Ali; Ghani, Eijaz; Naeem, Muhammad Ahsan; Imran, Muhammad; Iqbal, Naveed; Rehman, Sajjad-ur; Waruhiu, Cecilia; Shi, Zheng-Li | 2019 | 10.1016/j.ijid.2019.01.020, | Article | Detection of West Nile virus lineage 1 sequences in blood donors, Punjab Province, Pakistan | This study was performed to determine the presence of West Nile virus (WNV) in mosquito specimens and human blood donors in Pakistan. | Qualitative | Pakistan |
| Lingala MAL, Singh P, Verma P, Dhiman RC | 2020 | 10.1016/j.jiph.2019.11.017 | Article | Determining the cutoff of rainfall for Plasmodium falciparum malaria outbreaks in India | To determine the amount of rainfall required for malaria outbreaks and the lag period between outbreak and rainfall in different Indian climatic regions. | Quantitative | India |
| Daisy SS, Saiful Islam AKM, Akanda AS, Faruque ASG, Amin N, Jensen PKM | 2020 | 10.2166/wh.2020.133 | Article | Developing a forecasting model for cholera incidence in Dhaka megacity through time series climate data | Predicting cholera incidence; so that preparedness and emergency response plans can be taken into consideration in a more comprehensive way than at present | Qualitative | Bangladesh |
| Karim MJ, Haq R, Mableson HE, Sultan Mahmood ASM, Rahman M, Chowdhury SM, Rahman AKMF, Hafiz I, Betts H, Mackenzie C, Taylor MJ, Kelly-Hope LA | 2019 | 10.1371/journal.pntd.0007542 | Article | Developing the first national database and map of lymphatic filarasis clinical cases in Bangladesh: Another step closer to the elimination goals. | To describe the way in which the Bangladesh LF Programme worked to provide MMDP training to upazila and community clinic staff across all endemic areas to enable them to search, identify and report the number of clinical cases found in their community clinic catchment area | Quantitative | Bangladesh |
| Srividya A, Lall R, Ramaiah KD, Ramu K, Hoti SL, Pani SP, Das PK | 2000 | 10.1046/j.1365-3156.2000.00515.x | Article | Development of rapid assessment procedures for the delimitation of lymphatic filariasis-endemic areas | to develop alternate Rapid Assessment Procedures (RAP) for the delimitation of filarial endemic areas | Quantitative | India |
| Garg A, Dhiman RC, Bhattacharya S, Shukla PR | 2009 | 10.1007/s00267-008-9242-z | Article | Development, malaria and adaptation to climate change: a case study from India. | This article integrates climate change and developmental variables in articulating a framework for integrated impact assessment and adaptation responses, with malaria incidence in India as a case study | Quantitative | India |
| Verma, Preeti; Sarkar, Soma; Singh, Poonam; Dhiman, Ramesh C. | 2017 | 10.4103/ijmr.IJMR_426_16 | Article | Devising a method towards development of early warning tool for detection of malaria outbreak | The present study was aimed to generalize the theoretical structure of sine curve for detecting an outbreak so that a tool for early warning of malaria may be developed. | Mixed methods | India |
| Baig MA, Shaikh BT | 2012 | 10.1177/1010539510395377 | Article | Disease Surveillance System: A Mandatory Conduit for Effective Control of Infectious Diseases in Pakistan | To discuss the potentials and implications of replicating a surveillance system in other districts of Pakistan and analyze the barriers and constraints | Qualitative | Pakistan |
| Aziz S, Pakhtigian EL, Akanda AS, Jutla A, Huq A, Alam M, Ashan GU, Colwell RR | 2021 | 10.1016/j.socscimed.2021.113716, | Article | Does improved risk information increase the value of cholera prevention? An analysis of stated vaccine demand in slum areas of urban Bangladesh | Investigate demand for a reduction in cholera risk in two slum communities in Dhaka, Bangladesh—Mirpur and Karail—by estimating household willingness to pay (WTP) for a cholera vaccine. | Quantitative | Bangladesh |
| Nasr-Azadani F, Unnikrishnan A, Akanda A, Islam S, Alam M, Huq A, Jutla A, Colwell R | 2015 | 10.3354/cr01310 | Article | Downscaling river discharge to assess the effects of climate change on cholera outbreaks in the Bengal Delta | To determine the effect of climate change on river discharge and thereafter on endemic cholera in the Bengal Delta. | Quantitative | Bangladesh |
| Ramaiah KD, Vijay Kumar KN | 2000 | 10.1016/S0001-706X(00)00102-9 | Article | Effect of lymphatic filariasis on school children | Assessing the effect of lymphatic filariasis on school children | Quantitative | India |
| Lingala MAL | 2017 | 10.1016/j.jiph.2017.02.007, | Article | Effect of meteorological variables on Plasmodium vivax and Plasmodium falciparum malaria in outbreak prone districts of Rajasthan, India | To study the effect of meteorological variables on Plasmodium vivax and Plasmodium falciparum malaria outbreaks for the period of 2009-2012. | Quantitative | India |
| Kovats RS, Bouma MJ, Hajat S, Worrall E, Haines A | 2003 | 10.1016/S0140-6736(03)14695-8 | Article | El Niño and health. | Explore the effect of ENSO on cholera risk in Bangladesh, and malaria epidemics in parts of South Asia and South America has been well established | Quantitative | Bangladesh |
| Pramanik M, Singh P, Kumar G, Ojha VP, Dhiman RC | 2020 | 10.1186/s12889-020-09609-1 | Article | El Nino Southern Oscillation as an early warning tool for dengue outbreak in India | The study was undertaken to find out the link between El Niño, precipitation, and dengue cases, which could help in early preparedness for control of dengue. | Mixed methods | India |
| Pramanik, Malay; Singh, Poonam; Kumar, Gaurav; Ojha, V. P.; Dhiman, Ramesh C. | 2020 | 10.1186/s12889-020-09609-1 | Article | El Niño Southern Oscillation as an early warning tool for dengue outbreak in India. | The study was undertaken to find out the link between El Niño, precipitation, and dengue cases, which could help in early preparedness for control of dengue | Quantitative | India |
| Siddique AK, Nair GB, Alam M, Sack DA, Huq A, Nizam A, Longini IM, Qadri F, Faruque SM, Colwell RR, Ahmed S, Iqbal A, Bhuiyan NA, Sack RB | 2016 | 10.1017/S0950268809990550 | Article | El Tor cholera with severe disease: a new threat to Asia and beyond | To understand whether severe dehydration produced by the El Tor biotype was due to a shift from El Tor to classical CT or due to other factors | Quantitative | Bangladesh |
| Lohitharajah J, Malavige GN, Chua AJ, Ng ML, Arambepola C, Chang T | 2015 | 10.1186/s12879-015-1040-7 | Article | Emergence of human West Nile Virus infection in Sri Lanka | Report the first identification of human WNV infection in Sri Lanka in patients presenting with meningoencephalitis. | Quantitative | Sri Lanka |
| Khatun T, Chatterjee S | 2017 | 10.1093/trstmh/trx033, | Article | Emergence of West Nile virus in West Bengal, India: a new report | Explored the existence of WNV within undiagnosed samples to identify the emergence of a new public health problem. | Quantitative | India |
| Kumar S, Maurya VK, Saxena SK | 2020 | 10.1007/978-981-13-9197-2_5 | Article | Emerging and Re-emerging Water-Associated Infectious Diseases | Describing factors that can cause emergence of water-associated infectious diseases | Quantitative | India |
| Rodo X, Pascual M, Fuchs G, Faruque AS | 2020 | 10.1073/pnas.182203999 | Article | ENSO and cholera: a nonstationary link related to climate change? | To provide evidence for a change in the association of cholera dynamics and ENSO from the first to the last decades of the 20th century. | Qualitative | Bangladesh |
| Rao NV, Nagendra H | 2020 | 10.18520/cs/v119/i12/1919-1926 | Review | Epidemics and climate change in India | To synthesize evidence on the impact of climate on infectious diseases | Quantitative | India |
| Khatri V, Amdare N, Chauhan N, Togre N, Reddy MV, Hoti SL, Kalyanasundaram R | 2019 | 10.1007/s00436-019-06205-0, | Article | Epidemiological screening and xenomonitoring for human lymphatic filariasis infection in select districts in the states of Maharashtra and Karnataka, India | Surveyed select endemic regions within India to determine the status of LF infections | Quantitative | India |
| Shrestha SB, Pyakurel UR, Khanal M, Upadhyay M, Na-Bangchang K, Muhamad P | 2019 | 10.1108/JHR-10-2018-0124 | Article | Epidemiological situations and control strategies of vector-borne diseases in Nepal during 1998–2016 | investigate epidemiology and control strategies of the four priority vector-borne diseases (VBDs) in Nepal, i.e. malaria, Kala-azar (visceral leishmaniasis), lymphatic filariasis (LF) and dengue fever/dengue hemorrhagic fever | Quantitative | Nepal |
| Tsheten T, Gray DJ, Clements ACA, Wangdi K | 2021 | 10.1093/trstmh/traa158 | Review | Epidemiology and challenges of dengue surveillance in the WHO South-East Asia Region. | To describe the contemporary epidemiology of dengue and critically analyse the existing surveillance strategies in the region | Quantitative | South-East Asia |
| Khan AI, Rashid MM, Islam MT, Afrad MH, Salimuzzaman M, Hegde ST, Zion MMI, Khan ZH, Shirin T, Habib ZH, Khan IA, Begum YA, Azman AS, Rahman M, Clemens JD, Flora MS, Qadri F | 2020 | 10.1093/cid/ciz1075 | Article | Epidemiology of Cholera in Bangladesh: Findings From Nationwide Hospital-based Surveillance, 2014-2018 | Conducted systematic hospital-based cholera surveillance among diarrhea patients in 22 sites throughout Bangladesh from 2014 to 2018 | Quantitative | Bangladesh |
| Naseer M, Jamali T | 2014 | 08.2014/JCPSP.855860 | Review | Epidemiology, Determinants and Dynamics of Cholera in Pakistan: Gaps and Prospects for Future Research | To understand the epidemiology and to identify the possible determinants of cholera infection in Pakistan | Quantitative | Pakistan |
| M. S. CHAN, A. SRIVIDYA, R. A. NORMAN, S. P. PANI, K. D. RAMAIAH, P. VANAMAIL, E. MICHAEL, P. K. DAS, AND D. A. P. BUNDY | 1998 | 10.4269/ajtmh.1998.59.606 | Article | EPIFIL: A DYNAMIC MODEL OF INFECTION AND DISEASE IN LYMPHATIC FILARIASIS | Describe the development and validation of EPIFIL, a dynamic model of filariasis infection intensity and chronic disease. | Quantitative | India |
| Chaturvedi S, Dwivedi S | 2020 | 10.2166/wh.2020.148 | Article | Estimating the malaria transmission over the Indian subcontinent in a warming environment using a dynamical malaria model | The main aim of this paper is to investigate the spatio-temporal variability in malaria transmission patterns over the Indian | Mixed methods | India |
| Rutvisuttinunt W, Chinnawirotpisan P, Klungthong C, Shrestha SK, Thapa AB, Pant A, Yingst SL, Yoon IK, Fernandez S, Pavlin JA | 2014 | 10.1186/s12879-014-0606-0 | Article | Evidence of West Nile virus infection in Nepal | To identify known and unknown pathogens circulating in Nepal. Method | Quantitative | Nepal |
| Sirisena PD, Noordeen F | 2014 | 10.1016/j.ijid.2013.10.012 | Review | Evolution of dengue in Sri Lanka-changes in the virus, vector, and climate | To analyze the evolution of dengue in Sri Lanka—changes in the virus, vector, and climate | Quantitative | Sri Lanka |
| Srividya A, Das PK, Ramaiah KD, Grenfell BT, Michael E, Bundy DAP | 1994 | 10.1051/parasite/199401s1002 | Article | EXPOSURE AND THE DYNAMICS OF LYMPHATIC FILARIASIS INFECTION | To address the problem of how changes through time in the density of biting mosquitoes affect the acquirance of Wucbecheria bancrofti infection in young children | Qualitative | India |
| Banjara MR, Kroeger A, Huda MM, Kumar V, Gurung CK, Das ML, Rijal S, Das P, Mondal D | 2015 | 10.1093/trstmh/trv031 | Article | Feasibility of a combined camp approach for vector control together with active case detection of visceral leishmaniasis, post kala-azar dermal leishmaniasis, tuberculosis, leprosy and malaria in Bangladesh, India and Nepal: an exploratory study | To assess the feasibility, costs and results of a combination of active VL and PKDL case detection (including other fever diseases) and transmission reduction through vector control (bednet impregnation with slow release insecticide KOTAB 123, Bayer, Lyon, France) using the combined camp approach in Bangladesh, India and Nepal. | Qualitative | Bangladesh, India, and Nepal |
| Patil S, Pandya S | 2021 | 10.3389/fpubh.2021.798034 | Article | Forecasting Dengue Hotspots Associated With Variation in Meteorological Parameters Using Regression and Time Series Models | proposes a forecasting model for predicting dengue incidences considering climatic variability across nine cities of Maharashtra state of India over 10 years. | Quantitative | India |
| Ramaiah KD, Kumar KN, Ramu K, Pani SP, Das PK | 1997 | 10.1046/j.1365-3156.1997.d01-406.x | Article | Functional impairment caused by lymphatic filariasis in rural areas of South India | To assess the socio-economic impact of the Lymphatic filariasis | Quantitative | India |
| Dissanayake DMRB, Stephen C, Daniel S, Abeynayake P | 2012 | 10.5367/oa.2012.0091 | Article | Gap assessment of animal health legislation in sri lanka for emerging infectious disease preparedness | To review national, provincial and local animal health legislation in Sri Lanka to determine whether it supported EID preparedness | Quantitative | Sri Lanka |
| Ramasamy R, Surendran SN | 2012 | 10.3389/fphys.2012.00198 | Review | Global climate change and its potential impact on disease transmission by salinity-tolerant mosquito vectors in coastal zones | Provide an overview of the possible effects of global climate change and rising sea levels on mosquito-borne diseases in coastal zones, with dengue and malaria as particular examples | Quantitative | Sri Lanaka |
| Karunasena VM, Marasinghe M, Koo C, Amarasinghe S, Senaratne AS, Hasantha R, Hewavitharana M, Hapuarachchi HC, Herath HDB, Wickremasinghe R, Mendis KN, Fernando D, Ranaweera D | 2019 | 10.1186/s12936-019-2843-6 | Article | he first introduced malaria case reported from Sri Lanka after elimination: implications for preventing the re-introduction of malaria in recently eliminated countries | This study describes the probable index case and the introduced case of malaria, which was diagnosed, and the actions taken to curtail further onward spread of the disease. | Quantitative | Sri Lanka |
| Zohaib A, Saqib M, Beck C, Hussain MH, Lowenski S, Lecollinet S, Sial A, Asi MN, Mansoor MK, Saqalein M, Sajid MS, Ashfaq K, Muhammad G, Cao S | 2015 | 10.1017/S0950268814002878 | Article | High prevalence of West Nile virus in equines from the two provinces of Pakistan | The purpose of this study is to determine the status of WNV and JEV in the resident equine population from selected districts of two provinces [Punjab (PUNJ) and Khyber Pakhtunkhwa (KPK)] of Pakistan | Qualitative | Pakistan |
| Reiner RC, King AA, Emch M, Yunus M, Faruque AS, Pascual M | 2012 | 10.1073/pnas.1108438109 | Article | Highly localized sensitivity to climate forcing drives endemic cholera in a megacity | formulate a probabilistic model for cholera dynamics within the city of Dhaka | Quantitative | Bangladesh |
| Sarkar R, Kessler A, Mawkhlieng B, Sullivan SA, Wilson ML, Carlton JM, Albert S | 2021 | 10.1186/s12936-021-03982-x | Article | Household and individual level risk factors associated with declining malaria incidence in Meghalaya, India: implications for malaria elimination in low-endemic settings | To undertaken to better understand the epidemiology of malaria in northeast India. | Quantitative | India |
| Lahariya C, Tomar SS | 2011 | PMID: 21406730 | Review | How endemic countries can accelerate lymphatic filariasis elimination? An analytical review to identify strategic and programmatic interventions. | This review of literature and analysis was conducted to identify additional and sustainable strategies to accelerate LF elimination from endemic countries | Quantitative | India |
| Dhimal M, Bhandari D, Dhimal ML, Kafle N, Pyakurel P, Mahotra N, Akhtar S, Ismail T, Dhiman RC, Groneberg DA, Shrestha UB, Müller R | 2021 | 10.3389/fphys.2021.651189 | Review paper | Impact of Climate Change on Health and Well-Being of People in Hindu Kush Himalayan Region: A Narrative Review | Synthesize evidence on the impact of climate change on physical and mental well-being of the people living in the HKH and discuss the plausible health impacts on the residents that may be attributed to climate change. | Quantitative | Himalayan Region (Nepal, Bhutan, Afghanistan, Bangladesh, India, and Pakistan) |
| Rita Sharma | 2012 | https://doi.org/10.1659/MRD-JOURNAL-D-12-00068.1 | Article | Impacts on Human Health of Climate and Land Use Change in the Hindu Kush-Himalayan Region Overview of Available Information and Research Agenda | Review literature on the impacts of climate change and land use transition on human health in the HKH region | Mixed methods | Countries in Hindu Kush–Himalayan Region |
| Rajendran K, Sumi A, Bhattachariya MK, Manna B, Sur D, Kobayashi N, Ramamurthy T | 2011 | PMID: 21415487 | Article | Influence of relative humidity in Vibrio cholerae infection: a time series model | To find the influence of season on cholera using time Series Analysis and generalized linear model with special emphasis on relational impact and climate factors. | Quantitative | India |
| Wu J, Yunus M, Ali M, Escamilla V, Emch M | 2018 | 10.1016/j.envint.2018.08.012 | Review | Influences of heatwave, rainfall, and tree cover on cholera in Bangladesh | To examine the association between the risk of cholera and heatwaves as well as the modification effects of rainfall and tree cover. | Qualitative | Bangladesh |
| Devi RR, Raju V | 2018 | 10.4103/0972-9062.234622 | Article | Information technology in morbidity management of human lymphatic filariasis-A promising tool in global programme for elimination of lymphatic filariasis | | Qualitative |  |
| Taneja N, Mishra A, Batra N, Gupta P, Mahindroo J, Mohan B | 2020 | 10.1016/j.vaccine.2019.06.038 | Article | Inland cholera in freshwater environs of north India | Describe cholera epidemiology and explain the seasonality of cholera | Quantitative | India |
| Singh P, Yadav Y, Saraswat S, Dhiman RC | 2017 | 10.4103/0971-5916.193285 | Article | Intricacies of using temperature of different niches for assessing impact on malaria transmission | to understand the influence of different formats of temperature of different micro-niches on transmission of malaria for providing more realistic projections. | Quantitative | India |
| Akter R, Hu W, Naish S, Banu S, Tong S | 2017 | 10.1111/tmi.12868 | Review | Joint effects of climate variability and socioecological factors on dengue transmission: epidemiological evidence | To assess the epidemiological evidence on the joint effects of climate variability and socioecological factors on dengue transmission. | Quantitative | Asia, Latin America, North America |
| Ramaiah KD, Kumar KN, Ramu K | 1996 | 10.1046/j.1365-3156.1996.d01-84.x | Review | Knowledge and beliefs about transmission, prevention and control of lymphatic filariasis in rural areas of South India | Investigate into people’s knowledge, beliefs and behaviour in relation to lymphatic filariasis caused by Wuchereria bancrofti and transmitted by Culex guinquefasciatus in rural areas of Tamil Nadu, India | Mixed Method | India |
| Wahed T, Kaukab SS, Saha NC, Khan IA, Khanam F, Chowdhury F, Saha A, Khan AI, Siddik AU, Cravioto A, Qadri F, Uddin J | 2013 | 10.1186/1471-2458-13-242 | Article | Knowledge of, attitudes toward, and preventive practices relating to cholera and oral cholera vaccine among urban high-risk groups: findings of a cross-sectional study in Dhaka, Bangladesh | Assessed the knowledge of, attitudes toward, and preventive practices relating to cholera and oral cholera vaccine among an urban population residing in a high cholera-prone setting in Dhaka, Bangladesh. | Qualitative | Bangladesh |
| Kakarla SG, Caminade C, Mutheneni SR, Morse AP, Upadhyayula SM, Kadiri MR, Kumaraswamy S | 2019 | 10.1017/S0950268819000608 | Article | Lag effect of climatic variables on dengue burden in India | This study explores the relationship between El Niño Southern Oscillation (ENSO), the Indian Ocean Dipole (IOD) and dengue cases in India. | Mixed methods | India |
| Cash BA, Rodó X, Kinter JL | 2008 | 10.1175/2007JCLI2001.1 | Article | Links between Tropical Pacific SST and Cholera Incidence in Bangladesh: Role of the Eastern and Central Tropical Pacific | Investigate links between sea surface temperature in the central and eastern tropical Pacific and the regional climate of Bangladesh. | Quantitative | Bangladesh |
| Raju K, Jambulingam P, Sabesan S, Vanamail P | 2010 | 10.4103/0022-3859.68650 | Article | Lymphatic filariasis in India: Epidemiology and control measures | Discuss the epidemiology and current control strategy for filariasis; highlighting key issues, challenges and options in the implementation of the programme, and suggesting measures for mid-course corrections in the elimination strategy | Quantitative | India |
| Chandra G, Chatterjee SN, Das S, Sarkar N | 2007 | 10.1258/004947507781524737 | Article | Lymphatic filariasis in the coastal areas of Digha, West Bengal, India | To assess the epidemiology of lymphatic filariasis and the role of available mosquitoes as its vector in eight coastal villages around Digha, West Bengal | Quantitative | India |
| Sharma GM, Bhardwaj AR, Relwani NR, Dubey S | 2018 | 10.5704/MOJ.1803.016 | Article | Lymphatic Filariasis Presenting as a Swelling over the Upper Arm: A Case Report | Report a case of lymphatic filariasis in a 32-year old female who presented with a non-tender swelling over left upper arm | Quantitative | India |
| Sabesan S, Raju KH, Subramanian S, Srivastava PK, Jambulingam P | 2013 | 10.1089/vbz.2012.1238 | Article | Lymphatic filariasis transmission risk map of India, based on a geo-environmental risk model. | The purpose of the study was to create a filariasis transmission risk map for India based on GERM and using certain risk variables and determining whether risk of transmission exists in areas hitherto not surveyed. | Qualitative | India |
| Sharma R, Dutta AK | 2011 | 10.1007/s12098-011-0554-2 | Review | Malaria and National Vector Borne Disease Control Programme | Discusses the recent national drug policy for malaria and the rationale for its use. | Mixed Method | India |
| Yangzom T, Gueye CS, Namgay R, Galappaththy GN, Thimasarn K, Gosling R, Murugasampillay S, Dev V | 2012 | 10.1186/1475-2875-11-9 | Review paper | Malaria control in Bhutan: case study of a country embarking on elimination | This paper seeks to characterize the malaria programme of Bhutan over the last 10 years, exploring trends in the epidemiology of malaria, malaria control strategies and interventions, and the enabling and challenging conditions of Bhutan with emphasis on the endemic southern border and population migration | Quantitative | Bhutan |
| Dash AP, Valecha N, Anvikar AR, Kumar A | 2008 | 10.1007/s12038-008-0076-x | Article | Malaria in India: Challenges and opportunities | The estimation of true burden of malaria- administrative, financial, technical and operational challenges faced by the national programme | Quantitative | India |
| Das, Aparup; Anvikar, Anupkumar R.; Cator, Lauren J.; Dhiman, Ramesh C.; Eapen, Alex; Mishra, Neelima; Nagpal, Bhupinder N.; Nanda, Nutan; Raghavendra, Kamaraju; Read, Andrew F.; Sharma, Surya K.; Singh, Om P.; Singh, Vineeta; Sinnis, Photini; Srivastava, Harish C.; Sullivan, Steven A.; Sutton, Patrick L.; Thomas, Matthew B.; Carlton, Jane M.; Valecha, Neena | 2012 | 10.1016/j.actatropica.2011.11.008 | Article | Malaria in India: the center for the study of complex malaria in India. | Describe plans for a Center for the Study of Complex Malaria in India | Qualitative | India |
| Sarkar S, Singh P, Lingala MAL, Verma P, Dhiman RC | 2019 | 10.4081/gh.2019.767 | Article | Malaria risk map for India based on climate, ecology and geographical modelling | The study abides a holistic approach to risk mapping by including topographic, climatic and vegetation components into the framework of malaria risk modelling. | Quantitative | India |
| Gai PP, Mockenhaupt FP, Siegert K, Wedam J, Boloor A, Kulkarni SS, Rasalkar R, Kumar A, Jain A, Mahabala C, Gai P, Baliga S, Devi R, Shenoy D | 2018 | 10.1186/s12936-018-2462-7 | Article | Manifestation of malaria in Mangaluru, southern India | The present study aimed at providing a description of the manifestation of malaria at the largest governmental health facility in Mangaluru, the 900-bed Wenlock Hospital, and to specifically assess differences between P. vivax and P. falciparum mono-infections as well as mixed-species infections | Quantitative | India |
| Sabesan S, Palaniyandi M, Das PK, Michael E | 2000 | 10.1080/00034983.2000.11813582 | Article | Mapping of lymphatic filariasis in India | To assemble district-level endemicity maps of lymphatic filariasis in India, using a geographical information system and the best available survey data | Mixed methods | India |
| Rhee C, Gupta B, Lal B, Lim J, Wartel T, Lynch J, Sahastrabuddhe S | 2020 | 10.4103/1995-7645.278095 | Review | Mapping the high burden areas of cholera in Nepal for potential use of oral cholera vaccine: An analysis of data from publications and routine surveillance system | To assess the extent of existing published evidence on cholera and to characterize the epidemiologic data of cholera in Nepal | Mixed methods | Nepal |
| Nujum, Zinia T. | 2011 | 10.1016/j.inhe.2010.12.001 | Article | Coverage and compliance to mass drug administration for lymphatic filariasis elimination in a district of Kerala, India | To explore the association of sociodemographic variables with compliance and to find the reasons for noncompliance to the drug. | Quantitative | India |
| Jambulingam P, Subramanian S, de Vlas SJ, Vinubala C, Stolk WA | 2016 | 10.1186/s13071-016-1768-y | Article | Mathematical modelling of lymphatic filariasis elimination programmes in India: required duration of mass drug administration and post-treatment level of infection indicators. | To assess the required duration of MDA to achieve elimination and the associated 1-year post-treatment values of Mf and Ag prevalence associated with successful elimination, both for the community as a whole and for 6–7 year children only. | Quantitative | India |
| Rijal, Komal Raj; Adhikari, Bipin; Adhikari, Nabaraj; Dumre, Shyam Prakash; Banjara, Mayur Sharma; Shrestha, Upendra Thapa; Banjara, Megha Raj; Singh, Nihal; Ortegea, Leonard; Lal, Bibek Kumar; Das Thakur, Garib; Ghimire, Prakash | 2019 | 10.1186/s41182-019-0148-7 | Article | Micro-stratification of malaria risk in Nepal: implications for malaria control and elimination | The main objective of this study was to estimate the risk of malaria at Village Development Committee (VDC) level in Nepal based on disease, vector, parasite, and geography. | Quantitative | Nepal |
| Anwar A, Khan N, Ayub M, Nawaz F, Shah A, Flahault A | 2019 | 10.3390/ijerph16132296 | Article | Modeling and Predicting Dengue Incidence in Highly Vulnerable Countries using Panel Data Approach | This paper examines the effects of climate change and socio-economic variables on the incidence of dengue-borne diseases in some of the most highly vulnerable countries | Quantitative | Bangladesh, India, Philippines, Thailand, Myanmar and Zimbabwe |
| Bal S, Sodoudi S | 2020 | 10.1007/s00484-020-01918-9 | Article | Modeling and prediction of dengue occurrences in Kolkata, India, based on climate factors | To identify the relative contribution of the putative drivers responsible for dengue occurrences in Kolkata and project dengue incidences with respect to the future climate change. | Quantitative | India |
| Singh, Shikha | 2017 | 10.1007/s40808-017-0292-1 | Article | Modeling the effect of global warming on the spread of carrier dependent infectious diseases | | Quantitative |  |
| Irvine MA, Reimer LJ, Njenga SM, Gunawardena S, Kelly-Hope L, Bockarie M, Hollingsworth TD | 2015 | 10.1186/s13071-015-1152-3 | Article | Modelling strategies to break transmission of lymphatic filariasis--aggregation, adherence and vector competence greatly alter elimination. | Develop a new individual-based, stochastic mathematical model of the transmission of lymphatic filariasis | Quantitative | Sri Lanka |
| Chatterjee, Chandrajit; Sarkar, Ram Rup | 2009 | 10.1371/journal.pone.0004726 | Article | Multi-step polynomial regression method to model and forecast malaria incidence | Understand the causative factors such as age, sex, social factors, environmental variability etc. as well as underlying transmission dynamics of the disease for epidemiological research on malaria and its eradication. | Mixed methods | India |
| Perera M, Whitehead M, Molyneux D, Weerasooriya M, Gunatilleke G | 2007 | 10.1371/journal.pntd.0000128 | Article | Neglected Patients with a Neglected Disease? A Qualitative Study of Lymphatic Filariasis | This study aims to increase understanding of how this vulnerable, neglected group can be helped. | Quantitative | Sri Lanka |
| Gupta SS, Ganguly NK | 2020 | 10.1016/j.vaccine.2019.06.032 | Article | Opportunities and challenges for cholera control in India | Discuss the unique opportunity for India in the current scenario, to act against diseases like cholera and challenges that are anticipated in deployment of interventions due to suboptimal surveillance and shortage of vaccines. | Quantitative | India |
| Panda BB, Mohanty I, Rath A, Pradhan N, Hazra RK | 2019 | PMID: 33597483 | Article | Perennial malaria transmission and its association with rainfall at Kalahandi district of Odisha, Eastern India: A retrospective analysis | To understand the association of rainfall with perennial malaria transmission in Kalahandi district, Odisha | Quantitative | India |
| Krishna Kumari A, Harichandrakumar KT, Das LK, Krishnamoorthy K | 2005 | 10.1111/j.1365-3156.2005.01426.x | Article | Physical and psychosocial burden due to lymphatic filariasis as perceived by patients and medical experts. | To describe seven health states of LF based on patients’ perception and medical experts’ assessment. | Not mentioned | India |
| Vanamail P, Gunasekaran S | 2011 | 10.1007/s00038-010-0159-y | Article | Possible relationship among socio-economic determinants, knowledge and practices on lymphatic filariasis and implication for disease elimination in India | To assess the socio-economic determinants, knowledge and practices on lymphatic filariasis in India and discuss the implications for elimination. | Not mentioned | India |
| Roy, Manojit; Bouma, Menno; Dhiman, Ramesh C.; Pascual, Mercedes | 2015 | 10.1186/s12936-015-0937-3 | Article | Predictability of epidemic malaria under non-stationary conditions with process-based models combining epidemiological updates and climate variability | To distinguishing between the respective roles of (inter-annual) climate variability and intervention methods and improving predictability | Quantitative | India |
| Pascual M, Chaves LF, Cash B, Rodó X, Yunus M | 2008 | 10.3354/cr00730 | Article | Predicting endemic cholera: the role of climate variability and disease dynamics | Revisit semi-mechanistic model of cholera previously developed and parameterized for a time series of monthly cases in Matlab, Bangladesh, for the period from 1966 to 2002 | Quantitative | Bangladesh |
| Smith ME, Singh BK, Irvine MA, Stolk WA, Subramanian S, Hollingsworth TD, Michael E | 2017 | 10.1016/j.epidem.2017.02.006 | Article | Predicting lymphatic filariasis transmission and elimination dynamics using a multi-model ensemble framework | Report on the development of a first multi-model ensemble of three lymphatic filariasis (LF) models (EPIFIL, LYMFASIM, and TRANSFIL), and evaluate its predictive performance in comparison with that of the constituents using calibration and validation data from three case study sites | Quantitative | Southeast Asia |
| Cantey PT, Rao G, Rout J, Fox LM | 2008 | 10.1111/j.1365-3156.2009.02443.x | Article | Predictors of compliance with a mass drug administration programme for lymphatic filariasis in Orissa State, India 2008 | To assess the performance of an educational campaign to increase adherence to a mass-administered DEC regimen against lymphatic filariasis (LF) in Orissa, and to identify factors that could enhance future campaigns. | Quantitative | India |
| Wangdi K, Gatton ML, Kelly GC, Clements AC | 2014 | 10.1186/1475-2875-13-352 | Article | Prevalence of asymptomatic malaria and bed net ownership and use in Bhutan, 2013: a country earmarked for malaria elimination. | This study aimed to determine coverage, use and ownership of LLINs, as well as the prevalence of asymptomatic malaria at a single time-point, in four sub-districts of Bhutan | Quantitative | Bhutan |
| George S, Joy TM, Kumar A, Panicker KN, George LS, Raj M, Leelamoni K, Nair P | 2019 | 10.1007/s10903-018-0767-9, | Article | Prevalence of neglected tropical diseases (leishmaniasis and lymphatic filariasis) and malaria among a migrant labour settlement in kerala, india. | To assess the prevalence of NTDs (leishmaniasis and lymphatic filariasis) and malaria among a migrant labor settlement in Kochi Corporation of Ernakulam district in Kerala and also to study the associated risk factors for these diseases which will help in formulating policies to prevent transmission and morbidity and mortality associated with these diseases. | Quantitative | India |
| Md. Zafar Mahfooz Nomani and Rehana Parveen | 2020 | DOI: 10.1089/env.2019.0032 | Article | Prevention of chronic diseases in climate change scenario in india | To develop a comprehensive catalogue of climate change-associated health outcomes across the range of environments and populations for a better understanding of specific solutions | Qualitative | India |
| Kapa DR, Mohamed AJ | 2020 | 10.1093/inthealth/ihaa056 | Article | Progress and impact of 20 years of a lymphatic filariasis elimination programme in South-East Asia | Presents the progress and impact of lymphatic filariasis programme in the region. | Quantitative | South-East Asia |
| Alam, MS; Kabir, MM; Hossain, MS; Naher, S; Ferdous, NEN; Khan, WA; Mondal, D; Karim, J; Shamsuzzaman, AKM; Ahmed, BN; Islam, A; Haque, R | 2016 | 10.1186/s12936-016-1603-0 | Article | Reduction in malaria prevalence and increase in malaria awareness in endemic districts of Bangladesh | Measure the change in prevalence rate and people’s knowledge of malaria. | Qualitative | Bangladesh |
| Colombara DV, Faruque AS, Cowgill KD, Mayer JD | 2014 | 10.1186/1471-2334-14-440 | Article | Risk factors for diarrhea hospitalization in Bangladesh, 2000-2008: a case-case study of cholera and shigellosis | To identify cholera-specific risk factors distinct from shigellosis risk factors. | Quantitative | Bangladesh |
| Rajendran R, Sunish IP, Munirathinam A, Ashok Kumar V, Tyagi BK | 2010 | PMID: 20562816 | Article | Role of community empowerment in the elimination of lymphatic filariasis in south India | To determine the drug impact before and after each MDA | Quantitative | India |
| Pawar S, Kore M, Athalye A, Thombre PS | 2018 | 10.4103/ijhas.IJHAS_35_16 | Article | Seasonality of leptospirosis and its association with rainfall and humidity in Ratnagiri, Maharashtra | To study the seasonal pattern of leptospirosis cases and with rainfall and relative humidity and (2) to forecast the leptospirosis cases' occurrence based on the model. | Quantitative | India |
| Balakrishnan A, Thekkekare RJ, Sapkal G, Tandale BV | 2017 | 10.4103/ijmr.IJMR_1638_15 | Article | Seroprevalence of Japanese encephalitis virus & West Nile virus in Alappuzha district, Kerala | Undertake a population-based cross-sectional serosurvey of JEV and WNV in Alappuzha district. | Qualitative | India |
| Sarkar S, Gangare V, Singh P, Dhiman RC | 2019 | 10.3390/ijerph16183474 | Article | Shift in Potential Malaria Transmission Areas in India, Using the Fuzzy-Based Climate Suitability Malaria Transmission (FCSMT) Model under Changing Climatic Conditions | To assess, identify and map the potential effects of climate change on Plasmodium vivax (Pv) and Plasmodium falciparum (Pf) malaria transmission in India. | Quantitative | India |
| Dhimal M, Gautam I, Kreß A, Müller R, Kuch U | 2014 | 10.1371/journal.pntd.0003035 | Article | Spatio-temporal distribution of dengue and lymphatic filariasis vectors along an altitudinal transect in Central Nepal | To study the spatio-temporal distribution of dengue fever and lymphatic filariasis along an altitudinal transect in central Nepal. | Qualitative | Nepal |
| Dhimal M, Ahrens B, Kuch U | 2014 | 10.1186/s13071-014-0540-4 | Article | Species composition, seasonal occurrence, habitat preference and altitudinal distribution of malaria and other disease vectors in eastern Nepal. | to expand the current knowledge on the seasonal occurrence and altitudinal distribution of malaria and other disease vectors in eastern Nepal. | Quantitative | Nepal |
| Saha GK, Ganguly NK | 2021 | 10.1093/infdis/jiab436 | Article | Spread and Endemicity of Cholera in India: Factors Beyond the Numbers | Analyze how studies make sense of cholera transmission and spread in India from 1996 to 2015 | Not mentioned | India |
| Shrestha SL, Shrestha IL, Shrestha N, Joshi RD | 2017 | 10.1007/s10666-017-9547-5 | Article | Statistical Modeling of Health Effects on Climate-Sensitive Variables and Assessment of Environmental Burden of Diseases Attributable to Climate Change in Nepal | To quantify health-effect coefficients associated with climate-sensitive variables namely temperature, rainfall, relative humidity, and wind speed and estimate environmental burden of diseases attributed to temperature as the main climatic variable together with climate change in Nepal. | Mixed methods | Nepal |
| Saeed M, Faisal SM, Ahmad I, Kausar MA, Alam MJ, Khan S, Mustafa H | 2018 | 10.14715/cmb/2018.64.4.8 | Article | Status of lymphatic filariasis with progression of age and gender & eradication strategies: A survey among residents of Hardoi district of Uttar Pradesh, an endemic region of North India. | The aim was to see the impact of age and gender on various clinical forms of LF and in estimating its economic and social implications. | Quantitative | India |
| Nilmini T. G. A. Chandrasena, Ranjan Premaratna, Dilhani S. Samarasekera, Nilanthi R. de Silva | 2016 | 10.1093/trstmh/trw067 | Article | Surveillance for transmission of lymphatic filariasis in Colombo and Gampaha districts of Sri Lanka following mass drug administration | To assess the lymphatic filariasis situation, following mass drug administration. | Quantitative | Sri Lanka |
| K.D. Ramaiah, P. Vanamail | 2013 | https://doi.org/10.1093/trstmh/trt011 | Article | Surveillance of lymphatic filariasis after stopping ten years of mass drug administration in rural communities in south India | Provide information on the dynamics of the post-MDA threshold level lymphatic filariasis (LF) infection | Quantitative | India |
| Acharya B, Cao C, Xu M, Khanal L, Naeem S, Pandit S | 2018 | 10.3390/ijgi7070275 | Article | Temporal Variations and Associated Remotely Sensed Environmental Variables of Dengue Fever in Chitwan District, Nepal | To utilize epidemiological and earth observation data in Chitwan district, one of the frequent dengue outbreak areas of Nepal | Mixed methods | Nepal |
| Chowdhury FR, Ibrahim QSU, Bari MS, Alam MMJ, Dunachie SJ, Rodriguez-Morales AJ, Patwary MI | 2018 | 10.1371/journal.pone.0199579 | Article | The association between temperature, rainfall and humidity with common climate-sensitive infectious diseases in Bangladesh | To examined the association of temperature, humidity and rainfall with six common climate-sensitive infectious diseases in adults (malaria, diarrheal disease, enteric fever, encephalitis, pneumonia and bacterial meningitis) in northeastern Bangladesh | Qualitative | Bangladesh |
| Ramaiah KD, Das PK, Michael E, Guyatt HL | 2000 | 10.1016/S0169-4758(00)01643-4 | Review paper | The economic burden of lymphatic filariasis in india | Estimate the annual economic loss of lymphatic filariasis for India and discuss the implications | Quantitative | India |
| B.V. Babu , A.N. Nayak, K. Dhal, A.S. Acharya, P.K. Jangid, G. Mallic | 2001 | doi.org/10.1016/S0001-706X(02)00030-X | Article | The economic loss due to treatment costs and work loss to individuals with chronic lymphatic filariasis in rural communities of Orissa, India | to investigate the economic burden, in terms of treatment costs and loss of work to people affected with chronic lymphatic filariasis in rural communities of Orissa, Eastern India | Quantitative | India |
| Ehelepola ND, Ariyaratne K | 2015 | 10.3402/gha.v8.29359 | Article | The interrelationship between dengue incidence and diurnal ranges of temperature and humidity in a Sri Lankan city and its potential applications | To determine the correlation between dengue incidence and diurnal fluctuations of temperature and humidity in the Sri Lankan city of Kandy and to explore the possibilities of using that information for better control of dengue | Qualitative | Sri Lanka |
| Ali M, Emch M, Donnay JP, Yunus M, Sack RB | 2002 | 10.1016/s0277-9536(01)00230-1 | Article | The spatial epidemiology of cholera in an endemic area of Bangladesh | This paper defines high-risk areas of cholera based on environmental risk factors of the disease in an endemic area of Bangladesh | Quantitative | Bangladesh |
| Zeldenryk L, Gray M, Gordon S, Speare R, Hossain M | 2014 | 10.1007/s11136-013-0455-0 | Article | The use of focus groups to develop a culturally relevant quality of life tool for lymphatic filariasis in Bangladesh. | To conduct focus groups to operationalise the construct of quality of life (QOL) for people living with lymphatic filariasis (LF) in Bangladesh to develop culturally valid items for a Bangladeshi LF QOL tool | Qualitative | Bangladesh |
| Ali M, Kim DR, Yunus M, Emch M | 2013 | 10.3329/jhpn.v31i1.14744 | Article | Time series analysis of cholera in Matlab, Bangladesh, during 1988-2001 | examined the impact of in-situ climatic and marine environmental variability on cholera incidence in an endemic area of Bangladesh and developed a forecasting model for understanding the magnitude of incidence | Quantitative | Bangladesh |
| Garlapati R, Iniguez E, Serafim TD, Mishra PK, Rooj B, Sinha B, Valenzuela JG, Srikantiah S, Bern C, Kamhawi S | 2021 | 10.3389/fcimb.2021.641632 | Article | Towards a Sustainable Vector-Control Strategy in the Post Kala-Azar Elimination Era | discuss the successes and failures of previous and current vector-control strategies implemented to combat kala-azar in Bihar, India, and identify gaps in our understanding of vector transmission towards development of innovative tools to ensure sustained vector control in the post-elimination period. | Quantitative | India |
| Adimi F, Soebiyanto RP, Safi N, Kiang R | 2010 | 10.1186/1475-2875-9-125 | Article | Towards malaria risk prediction in Afghanistan using remote sensing | Understanding the role of environmental variables on malaria transmission | Quantitative | Afghanistan |
| Pilot, Eva; Roa, Ramana; Jena, Biranchi; Kauhl, Boris; Krafft, Thomas; Murthy, G. V. S. | 2017 | 10.3390/su9040604 | Article | Towards sustainable public health surveillance in india: using routinely collected electronic emergency medical service data for early warning of infectious diseases | To explore whether routinely collected emergency medical services (EMS) health data can improve sustainable infectious disease surveillance and early warning capacity and the need for improved surveillance systems for early warning of infectious diseases in India | Quantitative | India |
| Chaturvedi S, Dwivedi S | 2021 | 10.1007/s00484-021-02097-x | Article | Understanding the effect of climate change in the distribution and intensity of malaria transmission over India using a dynamical malaria model | To quantify and study the spatio-temporal intensity of malaria transmission and its variability in the context of climate change over different parts of India | Qualitative | India |
| Misslin R, Telle O, Daudé E, Vaguet A, Paul RE | 2016 | 10.1111/nyas.13084 | Review | Urban climate versus global climate change-what makes the difference for dengue? | Propose future necessary avenues of research, and underline the need to develop vector-control strategies pertinent to modern society. | Quantitative | India |
| Saulnier DD, Persson LÅ, Streatfield PK, Faruque AS, Rahman A | 2016 | 10.3402/gha.v9.30834 | Article | Using health and demographic surveillance for the early detection of cholera outbreaks: analysis of community- and hospital-based data from Matlab, Bangladesh | To determine whether increases in cholera in Matlab can be detected earlier by using HDSS diarrhea symptom data in a syndromic surveillance analysis, when compared to hospital admissions for cholera. | Mixed methods | Bangladesh |
| Sudeep AB, Mandar P, Ghodke YK, George RP, Gokhale MD | 2015 | PMID: 26418647 | Review paper | Vector competence of two Indian populations of Culex quinquefasciatus (Diptera: Culicidae) mosquitoes to three West Nile virus strains | To determine the growth kinetics and transmission mechanisms of three strains of WNV in two populations of Cx. quinquefasciatus. | Qualitative | India |
| Picado A, Dash AP, Bhattacharya S, Boelaert M | 2012 | PMID: 22885260 | Review | Vector control interventions for visceral leishmaniasis elimination initiative in South Asia, 2005-2010 | Present a review of studies published in the period 2005-2010 on the efficacy of different tools to control Phlebotomus argentipes. | Qualitative | South Asia |
| Rahman Z, Rahman MA, Rashid MU, Monira S, Johura FT, Mustafiz M, Bhuyian SI, Zohura F, Parvin T, Hasan K, Saif-Ur-Rahman KM, Islam NN, Sack DA, George CM, Alam M | 2018 | 10.3389/fpubh.2018.00238 | Article | Vibrio cholerae Transmits Through Water Among the Household Contacts of Cholera Patients in Cholera Endemic Coastal Villages of Bangladesh, 2015-2016 (CHoBI7 Trial) | investigate person to person and environmental transmission routes for cholera infection among household contacts of cholera cases | Qualitative | Bangladesh |
| Tamason CC, Tulsiani SM, Siddique AK, Hoque BA, Mackie Jensen PK | 2016 | 10.1186/s41043-016-0040-6 | Article | What is cholera? A preliminary study on caretakers' knowledge in Bangladesh | Aims to understand the current level of knowledge of cholera in female Bangladeshi caretakers, | Quantitative | Bangladesh |
