# Supplementary material for: Informing climate-health adaptation options through mapping the needs and potential for integrated climate-driven early warning forecasting systems in South Asia—A scoping review
Source: PLoS One. 2024 Oct 24;19(10):e0309757. doi: 10.1371/journal.pone.0309757 (PMC11500899; doi:10.1371/journal.pone.0309757)
Supplement: S2 Table — (DOCX) [file pone.0309757.s003.docx]

**Informing climate-health adaptation options through mapping the needs and potential for integrated climate-driven early warning forecasting systems in South Asia – a scoping review**

**S2 Table.** Annotated key studies reviewed by focal diseases of interests

| **Focal diseases** | **Doi** | **Country of report** |
| --- | --- | --- |
| Lymphatic Filariasis | 10.1371/journal.pone.0039970 | India |
| Lymphatic Filariasis | 10.1046/j.1365-3156.2000.00659.x | India |
| Lymphatic Filariasis | 10.1016/j.parint.2018.10.003 | Sri Lanka |
| Lymphatic Filariasis | 10.4103/0972-9062.313970 | India |
| Lymphatic Filariasis | 10.1016/j.trstmh.2005.07.016 | India |
| Lymphatic Filariasis; Malaria; Dengue; Visceral Leishmaniasis | 10.1007/978-981-10-7572-8_3 | Nepal |
| Leishmaniasis; Japanese Encephalitis | 10.20506/rst.30.3.2073 | India |
| Lymphatic Filariasis | 10.1093/trstmh/trv084 | Bangladesh |
| Lymphatic Filariasis | 10.1093/trstmh/tru057 | India |
| Lymphatic Filariasis | 10.1371/journal.pone.0039970 | India |
| Lymphatic Filariasis | 10.47276/lr.91.4.367 | India |
| Lymphatic Filariasis | 10.1371/journal.pntd.0007542 | Bangladesh |
| Lymphatic Filariasis | 10.1046/j.1365-3156.2000.00515.x | India |
| Lymphatic Filariasis | 10.1016/S0001-706X(00)00102-9 | India |
| Lymphatic Filariasis | 10.1007/s00436-019-06205-0 | India |
| Lymphatic Filariasis | 10.1051/parasite/199401s1002 | India |
| Lymphatic Filariasis | 10.1046/j.1365-3156.1997.d01-406.x | India |
| Lymphatic Filariasis | 10.4103/0972-9062.234622 | India |
| Lymphatic Filariasis | 10.1046/j.1365-3156.1996.d01-84.x | India |
| Lymphatic Filariasis | 10.4103/0022-3859.68650 | India |
| Lymphatic Filariasis | 10.1258/004947507781524737 | India |
| Lymphatic Filariasis | 10.5704/MOJ.1803.016 | India |
| Lymphatic Filariasis | 10.1089/vbz.2012.1238 | India |
| Lymphatic Filariasis | 10.1080/00034983.2000.11813582 | India |
| Lymphatic Filariasis | 10.1016/j.inhe.2010.12.001 | India |
| Lymphatic Filariasis | 10.1186/s13071-016-1768-y | India |
| Lymphatic Filariasis | 10.1007/s40808-017-0292-1 | India |
| Lymphatic Filariasis | 10.1186/s13071-015-1152-3 | Sri Lanka |
| Lymphatic Filariasis | 10.1371/journal.pone.0004726 | India |
| Lymphatic Filariasis | 10.1371/journal.pntd.0000128 | Sri Lanka |
| Lymphatic Filariasis | 10.1007/s00038-010-0159-y | India |
| Lymphatic Filariasis | 10.1016/j.epidem.2017.02.006 | Southeast Asia |
| Lymphatic Filariasis | 10.1111/j.1365-3156.2009.02443.x | India |
| Lymphatic Filariasis | 10.1093/inthealth/ihaa056 | South-East Asia |
| Lymphatic Filariasis | 10.14715/cmb/2018.64.4.8 | India |
| Lymphatic Filariasis | 10.1093/trstmh/trw067 | Sri Lanka |
| Lymphatic Filariasis | <https://doi.org/10.1093/trstmh/trt011> | India |
| Lymphatic Filariasis | 10.1016/S0169-4758(00)01643-4 | India |
| Lymphatic Filariasis | doi.org/10.1016/S0001-706X(02)00030-X | India |
| Lymphatic Filariasis | 10.4269/ajtmh.1998.59.606 | India |
| Lymphatic Filariasis | PMID: 21406730 | India |
| Lymphatic Filariasis | [PMID: 20562816](http://www.ncbi.nlm.nih.gov/pubmed/20562816) | India |
| Lymphatic Filariasis | 10.1111/j.1365-3156.2005.01426.x | India |
| Malaria | 10.4103/ijph.IJPH_306_17 | India |
| Malaria | 10.1007/s10668-021-01792-4 | India |
| Malaria | 10.1186/1475-2875-11-170 | Bangladesh |
| Malaria | 10.1186/s12936-021-03607-3 | India |
| Malaria | 10.1186/s41182-019-0148-7 | Nepal |
| Malaria | 10.54302/mausam.v72i2.611 | India |
| Malaria | 10.3402/iee.v6.30822 | South Asia |
| Malaria | 10.1186/s12936-016-1177-x | India |
| Malaria | PMC3612321 | India |
| Malaria | 10.1186/s12936-015-0697-0 | Sri Lanka |
| Malaria; Cholera | PMID: 31993386 | India; Bangladesh |
| malaria, Dengue, and Japanese encephalitis | 10.1007/s11069-019-03594-4 | India |
| Malaria | 10.1186/1475-2875-10-190 | India |
| Malaria, Dengue, Japanese encephalitis | 10.1111/1748-5967.12439 | India |
| Malaria | 10.1016/j.actatropica.2021.106040 | India |
| Malaria | 10.1016/j.jiph.2019.11.017 | India |
| Malaria | 10.1007/s00267-008-9242-z | India |
| Malaria | 10.4103/ijmr.IJMR_426_16 | India |
| Malaria | 10.1016/j.jiph.2017.02.007 | India |
| Malaria, Visceral leishmaniasis (Kala-azar), Lymphatic filariasis and Dengue fever/dengue haemorrhagic fever | 10.1108/JHR-10-2018-0124 | Nepal |
| Malaria | 10.2166/wh.2020.148 | India |
| Malaria | 10.1186/s12936-019-2843-6 | Sri Lanka |
| Malaria | 10.1186/s12936-021-03982-x | India |
| Malaria | 10.4103/0971-5916.193285 | India |
| Malaria | 10.1007/s12098-011-0554-2 | India |
| Malaria | 10.1186/1475-2875-11-9 | Bhutan |
| Malaria | 10.1007/s12038-008-0076-x | India |
| Malaria | 10.1016/j.actatropica.2011.11.008 | India |
| Malaria | 10.4081/gh.2019.767 | India |
| Malaria | 10.1186/s12936-018-2462-7 | India |
| Malaria | 10.1186/s41182-019-0148-7 | Nepal |
| Malaria | 10.1186/s12936-015-0937-3 | India |
| Malaria | 10.1186/1475-2875-13-352 | Bhutan |
| Malaria | 10.1186/s12936-016-1603-0 | Bangladesh |
| Malaria | 10.3390/ijerph16183474 | India |
| Malaria | 10.1186/s13071-014-0540-4 | Nepal |
| Malaria | 10.1371/journal.pone.0199579 | Bangladesh |
| Malaria | 10.1186/1475-2875-9-125 | Afghanistan |
| Malaria | 10.1007/s00484-021-02097-x | India |
| Malaria | 10.1038/s41467-022-28145-7 | India |
| Malaria; Dengue | 10.3389/fphys.2021.651189 | Himalayan Region (Nepal, Bhutan, Afghanistan, Bangladesh, India, and Pakistan) |
| Malaria; Dengue; Japanese encephalitis | <https://doi.org/10.1659/MRD-JOURNAL-D-12-00068.1> | Countries in Hindu Kush–Himalayan Region |
| Malaria | PMID: 33597483 | India |
| Malaria | DOI: 10.1089/env.2019.0032 | India |
| Malaria | 10.1002/hsr2.1775 | Afghanistan |
| Malaria; Dengue | 10.3389/fphys.2012.00198 | Sri Lanka |
| Cholera | 08.2014/JCPSP.855860 | Pakistan |
| Cholera | 10.1016/j.vaccine.2019.07.035 | Pakistan, India, Bangladesh, Nepal |
| Cholera | 10.3402/gha.v9.30834 | Bangladesh |
| Cholera | 10.1016/j.vaccine.2019.06.038 | India |
| Cholera | 10.1093/ije/dyw267 | Bangladesh |
| Cholera | 10.1016/j.ijid.2020.02.055 | Bangladesh |
| Cholera | 10.1126/science.289.5485.1766 | Bangladesh |
| Cholera | 10.1371/journal.pone.0172355 | Bangladesh |
| Cholera | 10.1016/j.ijid.2020.02.055 | Bangladesh |
| Cholera | PMID: 1500643 | Bangladesh |
| Cholera | 10.1016/j.advwatres.2016.11.013 | Bangladesh |
| Cholera | 10.1016/j.vaccine.2019.08.022 | Asia |
| Cholera | 10.1017/S0950268821001266 | India |
| Cholera | 10.2166/wh.2020.133 | Bangladesh |
| Cholera | 10.1016/j.socscimed.2021.113716 | Bangladesh |
| Cholera | 10.3354/cr01310 | Bangladesh |
| Cholera; Malaria | 10.1016/S0140-6736(03)14695-8 | Bangladesh |
| Cholera | 10.1017/S0950268809990550 | Bangladesh |
| Cholera | 10.1073/pnas.182203999 | Bangladesh |
| Cholera | 10.1093/cid/ciz1075 | Bangladesh |
| Cholera | 10.1073/pnas.1108438109 | Bangladesh |
| Cholera | 10.1016/j.envint.2018.08.012 | Bangladesh |
| Cholera | 10.1016/j.vaccine.2019.06.038 | India |
| Cholera | 10.1186/1471-2458-13-242 | Bangladesh |
| Cholera | 10.1175/2007JCLI2001.1 | Bangladesh |
| Cholera | 10.4103/1995-7645.278095 | Nepal |
| Cholera | 10.1016/j.vaccine.2019.06.032 | India |
| Cholera | 10.3354/cr00730 | Bangladesh |
| Cholera | 10.1186/1471-2334-14-440 | Bangladesh |
| Cholera | 10.1093/infdis/jiab436 | India |
| Cholera | 10.1016/s0277-9536(01)00230-1 | Bangladesh |
| Cholera | 10.1007/s11136-013-0455-0 | Bangladesh |
| Cholera | 10.3329/jhpn.v31i1.14744 | Bangladesh |
| Cholera | 10.3389/fpubh.2018.00238 | Bangladesh |
| Cholera | 10.1186/s41043-016-0040-6 | Bangladesh |
| Cholera | 08.2014/JCPSP.855860 | Pakistan |
| Cholera | PMID: 21415487 | India |
| Cholera | 10.1016/j.amsu.2022.104936 | India, Bangladesh and Nepal |
| Crimean-Congo haemorrhagic fever | https://doi.org/10.3855/jidc.15078 | Pakistan |
| Dengue | 10.1093/trstmh/traa093 | Bangladesh |
| Dengue | 10.1016/j.actatropica.2020.105337 | Nepal |
| Dengue | 10.1371/journal.pntd.0011820 | Bangladesh |
| Dengue, Lymphatic filariasis; Leishmaniasis, Malaria | 10.1016/j.accre.2021.05.003 | Hindu Kush Himalayan (HKH) region |
| Dengue | 10.1186/s40249-020-00717-z | Sri Lanka |
| Dengue fever; waterborne disease, Zika virus; Chikungunya | 10.3390/ijerph17228518 | Pakistan |
| Dengue | 10.1016/j.actatropica.2020.105337 | Nepal |
| Dengue | 10.1371/journal.pntd.0009624 | Sri Lanka |
| Dengue | 10.1016/j.scitotenv.2020.140336 | India |
| Dengue | 10.1016/j.envres.2020.110303 | Bangladesh |
| Dengue | 10.4103/jfmpc.jfmpc_716_19 | India |
| Dengue | 10.4103/0255-0857.115640 | India |
| Dengue | 10.3390/ijerph13111087 | Sri Lanka |
| Dengue | 10.1186/s40249-015-0075-8 | Sri Lanka |
| Dengue; Malaria; West Nile Fever | 10.1016/j.jiph.2017.12.006 | South Asia |
| Dengue | 10.3390/atmos12070905 | Bangladesh |
| Dengue | 10.7759/cureus.3398 | Bangladesh |
| Dengue | 10.3855/jidc.1017 | India |
| Dengue | 10.1002/rmv.1899 | Pakistan |
| Dengue | 10.1016/j.scitotenv.2020.140336 | India |
| Dengue | 10.7759/cureus.18500 | India |
| Dengue, Typhoid Legionellosis, Leptospirosis, Helicobacter pylori, Coxsackie viruses, Rotaviruses, Zika | 10.1007/978-981-13-9197-2_5 | India |
| Dengue | 10.1093/trstmh/traa158 | South-East Asia |
| Dengue | 10.1016/j.ijid.2013.10.012 | Sri Lanka |
| Dengue | 10.3389/fpubh.2021.798034 | India |
| Dengue | 10.1111/tmi.12868 | Asia |
| Dengue | 10.1017/S0950268819000608 | India |
| Dengue | 10.3390/ijerph16132296 | Bangladesh, India, Philippines, Thailand, Myanmar and Zimbabwe |
| Dengue | 10.1007/s00484-020-01918-9 | India |
| Dengue and Lymphatic filariasis | 10.1371/journal.pntd.0003035 | Nepal |
| Dengue, Japanese encephalitis, leishmaniasis, filariasis and malaria | 10.1007/s10666-017-9547-5 | Nepal |
| Dengue | 10.3390/ijgi7070275 | Nepal |
| Dengue | 10.3402/gha.v8.29359 | Sri Lanka |
| Dengue | 10.3390/su9040604 | India |
| Dengue | 10.1111/nyas.13084 | India |
| Dengue, leishmaniasis and lymphatic filariasis | 10.1371/journal.pone.0292723 | India |
| West Nile Virus | 10.1016/j.cimid.2013.10.006 | India |
| West Nile Virus | 10.1186/s13071-016-1948-9 | India |
| West Nile Virus | 10.1016/j.ijid.2019.01.020 | Pakistan |
| West Nile Virus | 10.1186/s12889-020-09609-1 | India |
| West Nile Virus | 10.1186/s12889-020-09609-1 | India |
| West Nile Virus | 10.1186/s12879-015-1040-7 | Sri Lanka |
| West Nile Virus | 10.1093/trstmh/trx033 | India |
| West Nile Virus | 10.1186/s12879-014-0606-0 | Nepal |
| West Nile virus; Japanese encephalitis virus | 10.1017/S0950268814002878 | Pakistan |
| West Nile Virus | PMID: 26418647 | India |
| Visceral leishmaniasis; Malaria | 10.1093/trstmh/trv031 | Bangladesh, India, and Nepal |
| Leishmaniasis and Lymphatic Filariasis | 10.1007/s10903-018-0767-9 | India |
| Visceral leishmaniasis (VL) | 10.3389/fcimb.2021.641632 | India |
| Visceral leishmaniasis (VL) | 10.1016/j.eswa.2023.121490 | India |
| Leishmaniasis | PMID: 22885260 | South Asia |
| Leishmaniasis | 10.1007/s10661-022-10274-7 | Sri Lanka |
| Leptospirosis | 10.4103/ijhas.IJHAS_35_16 | India |
| Leptospirosis | 10.1371/journal.pone.0275447 | India |
| Leptospirosis, Typhoid Legionellosis, Helicobacter pylori, Coxsackie viruses, Rotaviruses, Dengue, Zika | 10.1007/978-981-13-9197-2_5 | India |
| Scrub typhus | 10.4103/0972-9062.355958 | India |
| Japanese Encephalitis | 10.4103/ijmr.IJMR_1638_15 | India |
| Infectious disease | 10.1177/1010539510395377 | Pakistan |
| Unclear | 10.18520/cs/v119/i12/1919-1926 | India |
| Avian influenza | 10.5367/oa.2012.0091 | Sri Lanka |
| Vector-borne diseases | 10.1016/j.knosys.2023.110645 |  |
