# Supplementary material for: Informing climate-health adaptation options through mapping the needs and potential for integrated climate-driven early warning forecasting systems in South Asia—A scoping review
Source: PLoS One. 2024 Oct 24;19(10):e0309757. doi: 10.1371/journal.pone.0309757 (PMC11500899; doi:10.1371/journal.pone.0309757)
Supplement: S3 Table — (DOCX) [file pone.0309757.s004.docx]

**Informing climate-health adaptation options through mapping the needs and potential for integrated climate-driven early warning forecasting systems in South Asia – a scoping review**

**S3 Table. Overview of the main hydroclimate and health information systems and programs in the focal countries in south Asia**

| **ID** | **Country** | **Information platforms** | **Environmental Metrics/Data Captured** | **Status (In development/On-going?)** | **Time period** | **Spatial extent** | **Resolution** |
| --- | --- | --- | --- | --- | --- | --- | --- |
| 1 | India | Water quality monitoring- Central Pollution Control Board, India | Water temperature, pH, faecal coliform and other water quality metrics for Lakes, Rivers, Tanks and other water bodies in India. | No | 2013-2020 | National | Recording stations |
| 2 | India | Indian Water Resources Information System (WRIS), India | Precipitation, soil moisture, evapotranspiration, lake and reservoir storages. Forest/tree cover, land degradation, land cover and agro-climatic regions. | No | 2009- | National | District & Gridded |
| 3 | India | NHP Real Time Hydrological Information System (RTHIS), India* | Flood early warning system.  Drought warnings | Yes | NA | National | NA |
| 4 | India | NHP Real Time Hydrological Information System (RTHIS), India* | Flood early warning system.  Drought warnings | Yes | NA | National | NA |
| 5 | India | Climate Resilience Information System and Planning Tool for Mahatma Gandhi National Rural Employment Guarantee Scheme (The CRISP-M tool) | Watershed metrics, forest cover | Yes | NA | National | NA |
| 6 | Bangladesh | – Bangladesh Agro-meteorological Information Portal (BAMIS) of the Department of Agricultural Extension (DAE) | Brings in data from BMD and BWDB (see below 13 and 18)  Weather data – rainfall, temperature, humidity, clouds, wind speed and direction, sunshine hours  River water level  EO data (2013-2022) – (from NOAA STAR) vegetation health, smoothed temperature, moisture condition index, thermal index, rainfall, smoothed NDVI  Flood and drought information and advisories  Plant disease advisories (Wheat rust) | Yes, but operational | Developed through Weather and Climate Services regional project 2016- current (extended from 2021) | National | District and gridded  Observed, modelled |
| 7 | India | Flood monitoring web-based platform, CWC | Rainfall, river, water level | Yes | 1958- | National | Recording station |
| 8 | Bangladesh | Hydro-met monitoring and forecasting web-based portal, BWDB | Temperature, water level, rainfall | No | 1972- | National | District and gridded, recording station |
| 9 | Global | Global Flood Awareness system (GLOFAS) web platform | Stream flow | Yes | 2011- | Global | Gridded/ satellite earth observation |
| 10 | Sri Lanka | Hydro-met status and forecasts website, linked to social media | Water level | Yes | NA | National | NA |
| 11 | India | Flood Forecasting and Early Warning System | Rainfall, rivers in India, | Yes | 1945- | National | District & State Recording station/ forecasting stations |
| 12 | Global | Global future climate info provided by World Bank via interactive web platform | Precipitation and temperature | NA | 1901-2020 | Global (Pakistan, Sri Lanka, Bangladesh, Afghanistan, Nepal, Maldives) | Regional and gridded |
