# Supplementary material for: Informing climate-health adaptation options through mapping the needs and potential for integrated climate-driven early warning forecasting systems in South Asia—A scoping review
Source: PLoS One. 2024 Oct 24;19(10):e0309757. doi: 10.1371/journal.pone.0309757 (PMC11500899; doi:10.1371/journal.pone.0309757)
Supplement: S4 Table — (DOCX) [file pone.0309757.s005.docx]

**S4 Table. Overview of forecasting ability of National Meteorological Departments**

| **Name of Country/Met Department** | **Ability to forecast and give advanced warning on extreme weather events such as storms (e.g., monsoon) and heatwaves? *(Y/N)** | **Which Specific forecasting ability mentioned? specify,** | **Other forecasting abilities related to drivers such as floods and drought? (Y/N)** | **Which Specific forecasting ability mentioned? Specify.** | **Note** |
| --- | --- | --- | --- | --- | --- |
| India- India Meteorological Department | Y | Cyclones, thunderstorms, extreme Heavy rainfall, heat wave, cold wave, fog, cyclones (hourly bulleting)  ENSO Forecast (El Niño and La Niña) | Y | Flash flood, drought | Seasonal forecast, extended range forecast and short to medium range National capacity for cyclone forecast |
| Pakistan- Meteorological Department | Y | Heat waves, Rain-wind/thunderstorm, Heavy Snow fall, Dense fog, heavy rain | Y | Flash flood, drought | Much emphasis is on drought and flood. Little info on heavy thunderstorm |
| Bangladesh- Meteorological Department | Y | Cyclone, Heat wave, Extreme rainfall heavy fog. | Y | Drought, flood |  |
| Afghanistan- Meteorological Department | Y | Extreme temperature, Extreme/ Heavy rainfall/precipitation, thunderstorm, | Y | flash floods and snow | These weather events are stated on the Met Department website, but it is not clear if the country has the forecasting capabilities. |
| Sri Lanka- Department of Meteorology | Y | Heavy showers or thundershowers, strong winds and rough seas, forecasts of ENSO | NS | NS | No identified forecasting capabilities on the Met Department website and the reports. However, the stated weather events were mentioned on the sites |
| Bhutan- National Center for Hydrology and Meteorology | Y | Cyclone, extremely heavy rain | Y | Flood | Emphasis placed on Cyclonic storm and rainfall forecasting. Information derived from WMO |
| Nepal-Department of Hydrology and Meteorology | Y | No specific weather events mentioned | Y | Flood | Much emphasis on forecasting capabilities. WMO shows there is a forecasting capability but not specified. (Except flood showing on the Meteorology Department website) |
