# Supplementary material for: Informing climate-health adaptation options through mapping the needs and potential for integrated climate-driven early warning forecasting systems in South Asia—A scoping review
Source: PLoS One. 2024 Oct 24;19(10):e0309757. doi: 10.1371/journal.pone.0309757 (PMC11500899; doi:10.1371/journal.pone.0309757)
Supplement: S5 Table — (DOCX) [file pone.0309757.s006.docx]

**S5 Table. Overview of the health information systems and programs in the focal countries in south Asia**

| **Information platforms** | **Purpose** | **Open/**  **Proprietary resource** | **Ownership/ responsible authority & country** | **Developer** | **Sector/implementation scale** | **Associated Space time scale** | **Health Data Metrics/Data captured** | **Potential value for disease forecasting** |
| --- | --- | --- | --- | --- | --- | --- | --- | --- |
| Health Management Information System- India | To collate data on total number of publications on disease from India as proxy of funder or agency awards | Proprietary system | Ministry of Health and Family Welfare (MoHFW), India | Vyam Technologies | Public Health (state level) | No information provided | No data source mentioned | Japanese encephalitis, Leishmaniasis, Malaria, Tuberculosis, Typhoid and paratyphoid fevers |
| NIKSHAY**, India | To create a database of all TB patients and use this database for monitoring and research purposes | Proprietary system | National Informatics Centre (NIC), India | NIC | Public Health (national) | Daily | TB data | Tuberculosis |
| Integrated Health Information Platform (IHIP), India | The primary objective of IHIP is to enable the creation of standards compliant Electronic Health Records (EHRs) of the citizens on a pan-India basis along with the integration and interoperability of the EHRs through a comprehensive Health Information Exchange (HIE) as part of this centralized accessible platform. (Not fully operational) | Proprietary system | Ministry of Health and Family Welfare (MoHFW) | Not clear from website* | Public Health (national) | No information provided | Outbreak data | Guinea Worm, Yaws; Leptospirosis, Visceral Leishmaniasis, Japanese Encephalitis, Lymphatic Filariasis, Dengue |
| Web-base Malaria Management Information System, India | Monitoring and management of malaria cases and deaths in India | Proprietary system* | *Information not clear from the website | *Information not clear from the website | Public Health (national) | Monthly | No specific data mentioned | Malaria |
| Integrated Disease Surveillance Programme (IDSP), India | Strengthening of the disease surveillance system for epidemic-prone diseases to detect and respond to outbreaks | Open-source platform | National Centre for Disease Control (NCDC), Ministry of Health & Family Welfare (MoHFW), National Health Mission (NHM) | National Information Centre | Public Health (national) | weekly | suspected cases, presumptive cases, laboratory confirmed cases | Cholera, Enteric Fever, ADD, Viral Hepatitis A & E, Dengue, Leptospirosis and Chikungunya, West Nile Virus |
| National Animal Disease Referral Expert System (NADRES), India | Dynamic geographic information system and remote sensing-enabled expert system which is based on animal disease information collected and collated along with risk factor data of 652 (out of 735) districts of the country over a long period of time. | Proprietary system | National Institute of Veterinary Epidemiology and Disease Informatics (NIVEDI), Indian Council for Agricultural Research (ICAR), India | NIVEDI,  ICAR | Animal Health (national) | No information provided | Epidemiological data | 13 priority diseases  Anthrax, Babesiosis, Black Quarter, Bluetongue, Enterotoxaemia, Fasciolosis, Swine fever, Theileriosis, Trypanosomiasis |
| The Animal Disease Monitoring and Surveillance (ADMAS), India | Early warning of disease incidence or outbreaks and the alert system. Manages disease outbreaks and risk factor databases. | Proprietary system | NIVEDI, ICAR | The Indian Council of Agricultural Research (ICAR) | Animal Health (national) | No information provided | No data source provided | Animal Diseases |
|  |  |  |  |  |  |  |  |  |
| National Electronic Disease Surveillance System (eDEWS), India | An initiative that promotes the use of data and information system standards to advance the development of efficient, integrated, and interoperable  Surveillance systems at federal, provincial and district levels | Open access | Centre for Disease Control and Prevention | Open Health Marketplace | Public Health | No information provided | No data source provided | *Not clear from the website |
| Kala-azar Management Information System (KAMIS), India | Monitoring of disease trends, capture every case with accurate address of residence | Proprietary system | *Not clear from the website | *Not clear from the website | Public Health | No information provided | Kala-azar hospital cases | Visceral Leishmaniasis |
| Disease Early Warning System (DEWS) and Response monitors outbreaks of acute watery diarrhoea/ suspected cholera (2010–2014), Afghanistan | To contribute to the reduction of the morbidity, mortality and disability due to various health related problems in Afghanistan. | Open access | Ministry of Health, Pakistan | Establishment of Integrated- Pakistan | Public Health (national) | Weekly & Monthly | healthcare, hospitals and private clinics disease/ outbreak data | Diarrhoea, Cholera |
| Integrated Disease Surveillance and Response System (IDSRS) at provincial levels, linked with a public health laboratory in the provinces of Punjab and Sind- Pakistan | Health Information Systems in Pakistan collect data primarily from the vertical health programmes and District Health Information System (DHIS). | Open access | Ministry of National Health Services, Regulations and Coordination | Ministry of Health Khyber Pakhtunkhwa | Public Health (national) | No information provided | No health data mentioned | Acute Watery Diarrhoea / Cholera, Anthrax, Crimean Congo Haemorrhagic, Malaria, influenza, Leishmaniasis, dengue |
| Early Warning and Reporting System (EWARS), Nepal | Strengthen the flow of information on vector borne and other outbreak prone infectious diseases. A hospital-based sentinel surveillance system currently identified in 81 hospitals covering all 75 districts of Nepal. | *Not clear from the website | Ministry of Health Department of Health Services Epidemiology and Disease Control Division, Nepal | *Not clear from the website | Human Health (national) | Weekly | Hospital data, outbreak data | Cholera |
| Health Sector Information System (Nepal) | An integrated and comprehensive information system guided by National Strategy. The strategy is endorsed in 2007 by MoHP and being piloted in three districts Lalitpur, Parsa and Rupandehi. The implementation of HSIS is assigned to MD until the national health Information centre is established | Open-source platform | Ministry of Health and Population Department of Health Services, Nepal | Not clear from the website | Human Health (3 Districts) | No information provided | District and public health data |  |
| District Health Information System Bangladesh | Tool for collection, management, visualisation and generation of health information data | Open-source platform | Ministry of Health and Family Welfare (MoHFW), Bangladesh | DHIS2 (HISP, University of Oslo) | Human Health (national) | No information provided | Maternal mortality, new born and child mortality, communicable disease, non-communicable disease, universal health coverage data | Malaria, Visceral Leishmaniasis, Dengue, Cholera, Leptospirosis, Japanese encephalitis. |
| Shared Health Record (SHR) platform, Bangladesh | Making health information available across facilities. It also allows for more efficient care and better allocation of hospital resources. | *Not clear from the website | *Not clear from the website | India-based development | Human Health (rural communities) | No information provided | No data source mentioned |  |
