# Supplementary material for: Informing climate-health adaptation options through mapping the needs and potential for integrated climate-driven early warning forecasting systems in South Asia—A scoping review
Source: PLoS One. 2024 Oct 24;19(10):e0309757. doi: 10.1371/journal.pone.0309757 (PMC11500899; doi:10.1371/journal.pone.0309757)
Supplement: S6 Table — (DOCX) [file pone.0309757.s007.docx]

**S6 Table. Summary of studies where links were explored between driver and disease or national level health information systems feed into a study.** Environmental information systems were divided into two categories (National and Global)

| **Country** | **Disease** | **Driver** | **DOI** | **Health Information System** | **Environmental information System** | **EIS type** |
| --- | --- | --- | --- | --- | --- | --- |
| India | Malaria | Precipitation | 10.4081/gh.2019.767 | National Vector Borne Disease Control Program (NVBDCP) | IITM | National |
| India | Malaria | Natural cover change | 10.4081/gh.2019.767 | National Vector Borne Disease Control Program (NVBDCP) | Open Government Data | National |
| Bangladesh | Cholera | Warming | 10.3329/jhpn.v31i1.14744 | International Center for Diarrheal Disease Research (ICDDR, B) | Bangladesh Meteorological Department | National |
| Bangladesh | Cholera | Precipitation | 10.3329/jhpn.v31i1.14744 | International Center for Diarrheal Disease Research (ICDDR, B) | Bangladesh Meteorological Department | National |
| Bangladesh | Malaria | Temperature | 10.1186/1475-2875-11-170 | National health information system (Not Specified) | WORLDCLIM | Global |
| Bangladesh | Malaria | Precipitation | 10.1186/1475-2875-11-170 | National health information system (Not Specified) | WORLDCLIM | Global |
| Bangladesh | Malaria | Natural cover change | 10.1186/1475-2875-11-170 | National health information system (Not Specified) | GlobCover Land Cover | Global |
| Bangladesh | Cholera | - | PMID: 1500643 | International Center for Diarrheal Disease Research (ICDDR, B) | Not applicable | Not specified |
| India | Dengue | Temperature | 10.1016/j.scitotenv.2020.140336 | Integrated Disease Surveillance Program (IDSP); National Vector Borne Disease Control Program (NVBDCP) | Global Historical Climatology Network (GHCN) version 2; Climate Anomaly Monitoring System (GHCN-CAMS) | Global |
| India | Malaria | Rainfall | 10.2166/wh.2020.148 | National Vector Borne Disease Control Programme (NVBDCP) | CMIP5 models | Global |
| India | Malaria | Temperature | 10.2166/wh.2020.148 | National Vector Borne Disease Control Programme (NVBDCP) | CMIP5 models | Global |
| India | Malaria | - | 10.1186/s12936-021-03982-x | Meghalaya State Malaria Control Programme | Not applicable | Not specified |
| India | Cholera | - | 10.1016/j.vaccine.2019.06.038 | IDH-based surveillance | Not applicable | Not specified |
| India | Dengue | Temperature | 10.1017/S0950268819000608 | Integrated Disease Surveillance Program (IDSP) | NCEP-DOE 2 | Global |
| India | Dengue | Precipitation | 10.1017/S0950268819000608 | Integrated Disease Surveillance Program (IDSP) | Tropical Rainfall Measuring Mission (TRMM) | Global |
| India | Malaria | - | 10.1016/j.actatropica.2011.11.008 | Center for the Study of Complex Malaria in India (CSCMi); National Vector Borne Disease Control Programme (NVBDCP); | Not applicable | Not specified |
| Bangladesh | Cholera | Ocean climate change | 10.3354/cr00730 | International Center for Diarrheal Disease Research (ICDDR, B) | National Weather Service, Climate Prediction Centre, USA | Global |
| India | Visceral Leishmaniasis | - | 10.3389/fcimb.2021.641632 | Kala-azar Management Information System (KAMIS) and National Vector Borne Disease Control Program (NVBDCP) | Not applicable | Not specified |
