# Supplementary material for: Informing climate-health adaptation options through mapping the needs and potential for integrated climate-driven early warning forecasting systems in South Asia—A scoping review
Source: PLoS One. 2024 Oct 24;19(10):e0309757. doi: 10.1371/journal.pone.0309757 (PMC11500899; doi:10.1371/journal.pone.0309757)
Supplement: S7 Table — (DOCX) [file pone.0309757.s008.docx]

**Supporting information**

**S7 Table. Summary of all studies identified in the literature search**

| **ID** | **Article's Digital Object Identifier (DOI):** | **Article Title** | **Study eligible for inclusion**  **(Yes/No)** | **Reason for exclusion (if applicable)** |
| --- | --- | --- | --- | --- |
| 1 | 10.1016/j.envres.2020.110303 | COVID-19 pandemic, dengue epidemic, and climate change vulnerability in Bangladesh: Scenario assessment for strategic management and policy implications | Yes | Not applicable |
| 2 | 10.1007/s10668-021-01792-4 | A critical assessment of mosquito control and the influence of climate change on mosquito-borne disease epidemics | Yes | Not applicable |
| 3 |  | A focus of lymphatic filariasis in a tea garden worker community of central Assam | Yes | Not applicable |
| 4 | 10.1186/s12936-021-03607-3 | A model for malaria elimination based on learnings from the Malaria Elimination Demonstration Project, Mandla district, Madhya Pradesh | Yes | Not applicable |
| 5 | 10.4103/jfmpc.jfmpc_716_19 | A notorious vector-borne disease: Dengue fever, its evolution as public health threat. | Yes | Not applicable |
| 6 | 10.54302/mausam.v72i2.611 | A pilot study on assessing the effect of climate on the incidence of vector borne disease at Pune and Pimpri-Chinchwad area, Maharashtra | Yes | Not applicable |
| 7 | 10.1046/j.1365-3156.2000.00659.x | A programme to eliminate lymphatic filariasis in Tamil Nadu state, India: compliance with annual single-dose DEC mass treatment and some related operational aspects. | Yes | Not applicable |
| 8 | 10.3390/ijerph13111087 | A Spatial Hierarchical Analysis of the Temporal Influences of the El Nino-Southern Oscillation and Weather on Dengue in Kalutara District, Sri Lanka | Yes | Not applicable |
| 9 | 10.1186/s40249-015-0075-8 | A study of the correlation between dengue and weather in Kandy City, Sri Lanka (2003-2012) and lessons learned | Yes | Not applicable |
| 10 | 10.1016/j.parint.2018.10.003 | A surveillance system for lymphatic filariasis after its elimination in Sri Lanka. | Yes | Not applicable |
| 11 | 10.3402/iee.v6.30822 | A systematic review of knowledge, attitudes and beliefs about malaria among the South Asian population | Yes | Not applicable |
| 12 | 10.3389/fpubh.2018.00020 | Added to library on 10 Feb 2022 by You Human West Nile Virus Disease Outbreak in Pakistan, 2015-2016. | Yes | Not applicable |
| 13 | 10.1016/j.trstmh.2005.07.016, | Adverse reactions following mass drug administration during the Programme to Eliminate Lymphatic Filariasis in Orissa State, India | Yes | Not applicable |
| 14 | 10.1186/s12936-016-1177-x | Anopheles subpictus carry human malaria parasites in an urban area of Western India and may facilitate perennial malaria transmission | Yes | Not applicable |
| 15 | 10.54302/mausam.v70i4.264 | Application of spatial technology in malaria information infrastructure mapping with climate change perspective in Maharashtra, India | Yes | Not applicable |
| 16 | 10.4103/0255-0857.115640 | Assessing effect of climate on the incidence of dengue in Tamil Nadu | Yes | Not applicable |
| 17 | 10.1016/j.scitotenv.2021.148769 | Association between climate and infectious diseases among children in Varanasi city, India: A prospective cohort study. | Yes | Not applicable |
| 18 | 10.1007/s10393-013-0849-z | Association of weather and anthropogenic factors for transmission of Japanese encephalitis in an endemic area of India | Yes | Not applicable |
| 19 | 10.1016/j.meegid.2021.104987 | Asymptomatic malaria infection prevailing risks for human health and malaria elimination | Yes | Not applicable |
| 20 | 10.4103/0972-9062.313970 | Awareness and impact of Lymphatic Filariasis among school children in rural endemic areas of West Bengal, India | Yes | Not applicable |
| 21 | 10.4103/ijph.IJPH_306_17 | Basis of Science Policies for Infectious Disease Challenges in India | Yes | Not applicable |
| 22 | PMC3612321 | Battling malaria iceberg incorporating strategic reforms in achieving Millennium Development Goals & malaria elimination in India | Yes | Not applicable |
| 23 | 10.1016/j.ijid.2020.02.055 | Can cholera 'hotspots' be converted to cholera 'coldspots' in cholera endemic countries? The Matlab, Bangladesh experience | Yes | Not applicable |
| 24 | 10.1371/journal.pntd.0009624 | Changing epidemiology of dengue in Sri Lanka-Challenges for the future | Yes | Not applicable |
| 25 | 10.1007/978-981-10-7572-8_3 | Changing trend of infectious diseases in nepal | Yes | Not applicable |
| 26 | 10.1186/s12936-015-0697-0 | Characterization of imported malaria, the largest threat to sustained malaria elimination from Sri Lanka | Yes | Not applicable |
| 27 | 10.1016/j.cimid.2013.10.006 | Characterization of West Nile virus (WNV) isolates from Assam, India: Insights into the circulating WNV in northeastern India | Yes | Not applicable |
| 28 | 10.1186/1475-2875-11-170 | Characterizing the spatial and temporal variation of malaria incidence in Bangladesh, 2007 | Yes | Not applicable |
| 29 | 10.1093/ije/dyw267 | Cholera cases cluster in time and space in Matlab, Bangladesh: implications for targeted preventive interventions. | Yes | Not applicable |
| 30 | 10.1126/science.289.5485.1766 | Cholera dynamics and El Nino-Southern Oscillation | Yes | Not applicable |
| 31 | PMID: 1500643 | CHOLERA EPIDEMICS IN BANGLADESH - 1985-1991 | Yes | Not applicable |
| 32 | 10.1371/journal.pone.0172355 | Cholera forecast for Dhaka, Bangladesh, with the 2015-2016 El Niño: Lessons learned. | Yes | Not applicable |
| 33 | 10.1016/j.vaccine.2019.07.035, | Cholera in selected countries in Asia. | Yes | Not applicable |
| 34 | 10.1093/trstmh/traa093 | Climate change and dengue fever knowledge, attitudes and practices in Bangladesh: a social media-based cross-sectional survey | Yes | Not applicable |
| 35 | 10.1016/j.actatropica.2020.105337 | Climate change and infectious disease research in Nepal: Are the available prerequisites supportive enough to researchers? | Yes | Not applicable |
| 36 | PMID: 31993386 | Climate Change and Infectious Diseases: Evidence from Highly Vulnerable Countries | Yes | Not applicable |
| 37 | 10.1016/j.accre.2021.05.003 | Climate change and its association with the expansion of vectors and vector-borne diseases in the Hindu Kush Himalayan region: A systematic synthesis of the literature | Yes | Not applicable |
| 38 | 10.1007/s11069-019-03594-4 | Climate change and public health: a study of vector-borne diseases in Odisha, India | Yes | Not applicable |
| 39 | 10.20506/rst.30.3.2073 | Climate change and water resources of Himalayan region—review of impacts and implication | Yes | Not applicable |
| 40 | 10.1186/s40249-020-00717-z | Climate change induced vulnerability and adaption for dengue incidence in Colombo and Kandy districts: the detailed investigation in Sri Lanka. | Yes | Not applicable |
| 41 | 10.3390/ijerph17228518 | Climate Change, Water Quality and Water-Related Challenges: A Review with Focus on Pakistan | Yes | Not applicable |
| 42 | 10.1186/1475-2875-10-190 | Climate forcing and desert malaria: the effect of irrigation | Yes | Not applicable |
| 43 | 10.1016/j.jiph.2017.12.006 | Climate patterns and mosquito-borne disease outbreaks in South and Southeast Asia | Yes | Not applicable |
| 44 | 10.3390/atmos12070905 | Climate Variability, Dengue Vector Abundance and Dengue Fever Cases in Dhaka, Bangladesh: A Time-Series Study | Yes | Not applicable |
| 45 | 10.1016/j.advwatres.2016.11.013 | Climate-driven endemic cholera is modulated by human mobility in a megacity | Yes | Not applicable |
| 46 | 10.1093/trstmh/trv084 | Clinical case estimates of lymphatic filariasis in an endemic district of Bangladesh after a decade of mass drug administration | Yes | Not applicable |
| 47 | 10.1016/j.vaccine.2019.08.022 | Contrasts, contradictions and control of cholera | Yes | Not applicable |
| 48 | 10.7759/cureus.3398 | Correlates of Climate Variability and Dengue Fever in Two Metropolitan Cities in Bangladesh | Yes | Not applicable |
| 49 | 10.1093/trstmh/tru057 | Coverage of, and compliance with, mass drug administration under the programme to eliminate lymphatic filariasis in India: a systematic review | Yes | Not applicable |
| 50 | 10.1111/1748-5967.12439 | Current trends in large‐scale viral surveillance methods in mosquitoes | Yes | Not applicable |
| 51 | 10.1371/journal.pone.0039970 | Data base management system for lymphatic filariasis--a neglected tropical disease. | Yes | Not applicable |
| 52 | 10.1017/S0950268821001266 | Decades of cholera in Odisha, India (1993-2015): lessons learned and the ways forward | Yes | Not applicable |
| 53 | 10.1016/j.actatropica.2021.106040 | Defeating malaria in the North-East region: the forerunner for malaria elimination in India. | Yes | Not applicable |
| 54 | 10.3855/jidc.1017 | Dengue fever in the Indian Subcontinent: an overview | Yes | Not applicable |
| 55 | 10.1002/rmv.1899 | Dengue fever virus in Pakistan: effects of seasonal pattern and temperature change on distribution of vector and virus. | Yes | Not applicable |
| 56 | 10.1016/j.scitotenv.2020.140336 | Dengue situation in India: Suitability and transmission potential model for present and projected climate change scenarios | Yes | Not applicable |
| 57 | 10.7759/cureus.18500 | Dengue: A neglected disease of concern | Yes | Not applicable |
| 58 | 10.47276/lr.91.4.367 | Depression and anxiety in people affected by leprosy and lymphatic filariasis: a cross-sectional study in four States in India | Yes | Not applicable |
| 59 | 10.1186/s13071-016-1948-9 | Detection of West Nile virus in six mosquito species in synchrony with seroconversion among sentinel chickens in India. | Yes | Not applicable |
| 60 | 10.1016/j.ijid.2019.01.020, | Detection of West Nile virus lineage 1 sequences in blood donors, Punjab Province, Pakistan | Yes | Not applicable |
| 61 | 10.1016/j.jiph.2019.11.017 | Determining the cutoff of rainfall for Plasmodium falciparum malaria outbreaks in India | Yes | Not applicable |
| 62 | 10.2166/wh.2020.133 | Developing a forecasting model for cholera incidence in Dhaka megacity through time series climate data | Yes | Not applicable |
| 63 | 10.1371/journal.pntd.0007542 | Developing the first national database and map of lymphatic filarasis clinical cases in Bangladesh: Another step closer to the elimination goals. | Yes | Not applicable |
| 64 | 10.1046/j.1365-3156.2000.00515.x | Development of rapid assessment procedures for the delimitation of lymphatic filariasis-endemic areas | Yes | Not applicable |
| 65 | 10.1007/s00267-008-9242-z | Development, malaria and adaptation to climate change: a case study from India. | Yes | Not applicable |
| 66 | 10.4103/ijmr.IJMR_426_16 | Devising a method towards development of early warning tool for detection of malaria outbreak | Yes | Not applicable |
| 67 | 10.1177/1010539510395377 | Disease Surveillance System: A Mandatory Conduit for Effective Control of Infectious Diseases in Pakistan | Yes | Not applicable |
| 68 | 10.1016/j.socscimed.2021.113716, | Does improved risk information increase the value of cholera prevention? An analysis of stated vaccine demand in slum areas of urban Bangladesh | Yes | Not applicable |
| 69 | 10.3354/cr01310 | Downscaling river discharge to assess the effects of climate change on cholera outbreaks in the Bengal Delta | Yes | Not applicable |
| 70 | 10.1016/S0001-706X(00)00102-9 | Effect of lymphatic filariasis on school children | Yes | Not applicable |
| 71 | 10.1016/j.jiph.2017.02.007, | Effect of meteorological variables on Plasmodium vivax and Plasmodium falciparum malaria in outbreak prone districts of Rajasthan, India | Yes | Not applicable |
| 72 | 10.1016/S0140-6736(03)14695-8 | El Niño and health. | Yes | Not applicable |
| 73 | 10.1186/s12889-020-09609-1 | El Nino Southern Oscillation as an early warning tool for dengue outbreak in India | Yes | Not applicable |
| 74 | 10.1186/s12889-020-09609-1 | El Niño Southern Oscillation as an early warning tool for dengue outbreak in India. | Yes | Not applicable |
| 75 | 10.1017/S0950268809990550 | El Tor cholera with severe disease: a new threat to Asia and beyond | Yes | Not applicable |
| 76 | 10.1186/s12879-015-1040-7 | Emergence of human West Nile Virus infection in Sri Lanka | Yes | Not applicable |
| 77 | 10.1093/trstmh/trx033, | Emergence of West Nile virus in West Bengal, India: a new report | Yes | Not applicable |
| 78 | 10.1007/978-981-13-9197-2_5 | Emerging and Re-emerging Water-Associated Infectious Diseases | Yes | Not applicable |
| 79 | 10.1073/pnas.182203999 | ENSO and cholera: a nonstationary link related to climate change? | Yes | Not applicable |
| 80 | 10.18520/cs/v119/i12/1919-1926 | Epidemics and climate change in India | Yes | Not applicable |
| 81 | 10.1007/s00436-019-06205-0, | Epidemiological screening and xenomonitoring for human lymphatic filariasis infection in select districts in the states of Maharashtra and Karnataka, India | Yes | Not applicable |
| 82 | 10.1108/JHR-10-2018-0124 | Epidemiological situations and control strategies of vector-borne diseases in Nepal during 1998–2016 | Yes | Not applicable |
| 83 | 10.1093/trstmh/traa158 | Epidemiology and challenges of dengue surveillance in the WHO South-East Asia Region. | Yes | Not applicable |
| 84 | 10.1093/cid/ciz1075 | Epidemiology of Cholera in Bangladesh: Findings From Nationwide Hospital-based Surveillance, 2014-2018 | Yes | Not applicable |
| 85 | 08.2014/JCPSP.855860 | Epidemiology, Determinants and Dynamics of Cholera in Pakistan: Gaps and Prospects for Future Research | Yes | Not applicable |
| 86 | 10.4269/ajtmh.1998.59.606 | EPIFIL: A DYNAMIC MODEL OF INFECTION AND DISEASE IN LYMPHATIC FILARIASIS | Yes | Not applicable |
| 87 | 10.2166/wh.2020.148 | Estimating the malaria transmission over the Indian subcontinent in a warming environment using a dynamical malaria model | Yes | Not applicable |
| 88 | 10.1186/s12879-014-0606-0 | Evidence of West Nile virus infection in Nepal | Yes | Not applicable |
| 89 | 10.1016/j.ijid.2013.10.012 | Evolution of dengue in Sri Lanka-changes in the virus, vector, and climate | Yes | Not applicable |
| 90 | 10.1051/parasite/199401s1002 | EXPOSURE AND THE DYNAMICS OF LYMPHATIC FILARIASIS INFECTION | Yes | Not applicable |
| 91 | 10.1093/trstmh/trv031 | Feasibility of a combined camp approach for vector control together with active case detection of visceral leishmaniasis, post kala-azar dermal leishmaniasis, tuberculosis, leprosy and malaria in Bangladesh, India and Nepal: an exploratory study | Yes | Not applicable |
| 92 | 10.3389/fpubh.2021.798034 | Forecasting Dengue Hotspots Associated With Variation in Meteorological Parameters Using Regression and Time Series Models | Yes | Not applicable |
| 93 | 10.1046/j.1365-3156.1997.d01-406.x | Functional impairment caused by lymphatic filariasis in rural areas of South India | Yes | Not applicable |
| 94 | 10.5367/oa.2012.0091 | Gap assessment of animal health legislation in sri lanka for emerging infectious disease preparedness | Yes | Not applicable |
| 95 | 10.3389/fphys.2012.00198 | Global climate change and its potential impact on disease transmission by salinity-tolerant mosquito vectors in coastal zones | Yes | Not applicable |
| 96 | 10.1186/s12936-019-2843-6 | he first introduced malaria case reported from Sri Lanka after elimination: implications for preventing the re-introduction of malaria in recently eliminated countries | Yes | Not applicable |
| 97 | 10.1017/S0950268814002878 | High prevalence of West Nile virus in equines from the two provinces of Pakistan | Yes | Not applicable |
| 98 | 10.1073/pnas.1108438109 | Highly localized sensitivity to climate forcing drives endemic cholera in a megacity | Yes | Not applicable |
| 99 | 10.1186/s12936-021-03982-x | Household and individual level risk factors associated with declining malaria incidence in Meghalaya, India: implications for malaria elimination in low-endemic settings | Yes | Not applicable |
| 100 | PMID: 21406730 | How endemic countries can accelerate lymphatic filariasis elimination? An analytical review to identify strategic and programmatic interventions. | Yes | Not applicable |
| 101 | 10.3389/fphys.2021.651189 | Impact of Climate Change on Health and Well-Being of People in Hindu Kush Himalayan Region: A Narrative Review | Yes | Not applicable |
| 102 | <https://doi.org/10.1659/MRD-JOURNAL-D-12-00068.1> | Impacts on Human Health of Climate and Land Use Change in the Hindu Kush-Himalayan Region Overview of Available Information and Research Agenda | Yes | Not applicable |
| 103 | PMID: 21415487 | Influence of relative humidity in Vibrio cholerae infection: a time series model | Yes | Not applicable |
| 104 | 10.1016/j.envint.2018.08.012 | Influences of heatwave, rainfall, and tree cover on cholera in Bangladesh | Yes | Not applicable |
| 105 | 10.4103/0972-9062.234622 | Information technology in morbidity management of human lymphatic filariasis-A promising tool in global programme for elimination of lymphatic filariasis | Yes | Not applicable |
| 106 | 10.1016/j.vaccine.2019.06.038 | Inland cholera in freshwater environs of north India | Yes | Not applicable |
| 107 | 10.4103/0971-5916.193285 | Intricacies of using temperature of different niches for assessing impact on malaria transmission | Yes | Not applicable |
| 108 | 10.1111/tmi.12868 | Joint effects of climate variability and socioecological factors on dengue transmission: epidemiological evidence | Yes | Not applicable |
| 109 | 10.1046/j.1365-3156.1996.d01-84.x | Knowledge and beliefs about transmission, prevention and control of lymphatic filariasis in rural areas of South India | Yes | Not applicable |
| 110 | 10.1186/1471-2458-13-242 | Knowledge of, attitudes toward, and preventive practices relating to cholera and oral cholera vaccine among urban high-risk groups: findings of a cross-sectional study in Dhaka, Bangladesh | Yes | Not applicable |
| 111 | 10.1017/S0950268819000608 | Lag effect of climatic variables on dengue burden in India | Yes | Not applicable |
| 112 | 10.1175/2007JCLI2001.1 | Links between Tropical Pacific SST and Cholera Incidence in Bangladesh: Role of the Eastern and Central Tropical Pacific | Yes | Not applicable |
| 113 | 10.4103/0022-3859.68650 | Lymphatic filariasis in India: Epidemiology and control measures | Yes | Not applicable |
| 114 | 10.1258/004947507781524737 | Lymphatic filariasis in the coastal areas of Digha, West Bengal, India | Yes | Not applicable |
| 115 | 10.5704/MOJ.1803.016 | Lymphatic Filariasis Presenting as a Swelling over the Upper Arm: A Case Report | Yes | Not applicable |
| 116 | 10.1089/vbz.2012.1238 | Lymphatic filariasis transmission risk map of India, based on a geo-environmental risk model. | Yes | Not applicable |
| 117 | 10.1007/s12098-011-0554-2 | Malaria and National Vector Borne Disease Control Programme | Yes | Not applicable |
| 118 | 10.1186/1475-2875-11-9 | Malaria control in Bhutan: case study of a country embarking on elimination | Yes | Not applicable |
| 119 | 10.1007/s12038-008-0076-x | Malaria in India: Challenges and opportunities | Yes | Not applicable |
| 120 | 10.1016/j.actatropica.2011.11.008 | Malaria in India: the center for the study of complex malaria in India. | Yes | Not applicable |
| 121 | 10.4081/gh.2019.767 | Malaria risk map for India based on climate, ecology and geographical modelling | Yes | Not applicable |
| 122 | 10.1186/s12936-018-2462-7 | Manifestation of malaria in Mangaluru, southern India | Yes | Not applicable |
| 123 | 10.1080/00034983.2000.11813582 | Mapping of lymphatic filariasis in India | Yes | Not applicable |
| 124 | 10.4103/1995-7645.278095 | Mapping the high burden areas of cholera in Nepal for potential use of oral cholera vaccine: An analysis of data from publications and routine surveillance system | Yes | Not applicable |
| 125 | 10.1016/j.inhe.2010.12.001 | to estimate the coverage and compliance of MDA for 2007 in Thiruvananthapuram district of Kerala, India; to explore the association of sociodemographic variables with compliance and to find the reasons for noncompliance to the drug. | Yes | Not applicable |
| 126 | 10.1186/s13071-016-1768-y | Mathematical modelling of lymphatic filariasis elimination programmes in India: required duration of mass drug administration and post-treatment level of infection indicators. | Yes | Not applicable |
| 127 | 10.1186/s41182-019-0148-7 | Micro-stratification of malaria risk in Nepal: implications for malaria control and elimination | Yes | Not applicable |
| 128 | 10.3390/ijerph16132296 | Modeling and Predicting Dengue Incidence in Highly Vulnerable Countries using Panel Data Approach | Yes | Not applicable |
| 129 | 10.1007/s00484-020-01918-9 | Modeling and prediction of dengue occurrences in Kolkata, India, based on climate factors | Yes | Not applicable |
| 130 | 10.1007/s40808-017-0292-1 | Modeling the effect of global warming on the spread of carrier dependent infectious diseases | Yes | Not applicable |
| 131 | 10.1186/s13071-015-1152-3 | Modelling strategies to break transmission of lymphatic filariasis--aggregation, adherence and vector competence greatly alter elimination. | Yes | Not applicable |
| 132 | 10.1371/journal.pone.0004726 | Multi-step polynomial regression method to model and forecast malaria incidence | Yes | Not applicable |
| 133 | 10.1371/journal.pntd.0000128 | Neglected Patients with a Neglected Disease? A Qualitative Study of Lymphatic Filariasis | Yes | Not applicable |
| 134 | 10.1016/j.vaccine.2019.06.032 | Opportunities and challenges for cholera control in India | Yes | Not applicable |
| 135 | PMID: 33597483 | Perennial malaria transmission and its association with rainfall at Kalahandi district of Odisha, Eastern India: A retrospective analysis | Yes | Not applicable |
| 136 | 10.1111/j.1365-3156.2005.01426.x | Physical and psychosocial burden due to lymphatic filariasis as perceived by patients and medical experts. | Yes | Not applicable |
| 137 | 10.1007/s00038-010-0159-y | Possible relationship among socio-economic determinants, knowledge and practices on lymphatic filariasis and implication for disease elimination in India | Yes | Not applicable |
| 138 | 10.1186/s12936-015-0937-3 | Predictability of epidemic malaria under non-stationary conditions with process-based models combining epidemiological updates and climate variability | Yes | Not applicable |
| 139 | 10.3354/cr00730 | Predicting endemic cholera: the role of climate variability and disease dynamics | Yes | Not applicable |
| 140 | 10.1016/j.epidem.2017.02.006 | Predicting lymphatic filariasis transmission and elimination dynamics using a multi-model ensemble framework | Yes | Not applicable |
| 141 | 10.1111/j.1365-3156.2009.02443.x | Predictors of compliance with a mass drug administration programme for lymphatic filariasis in Orissa State, India 2008 | Yes | Not applicable |
| 142 | 10.1186/1475-2875-13-352 | Prevalence of asymptomatic malaria and bed net ownership and use in Bhutan, 2013: a country earmarked for malaria elimination. | Yes | Not applicable |
| 143 | 10.1007/s10903-018-0767-9, | Prevalence of neglected tropical diseases (leishmaniasis and lymphatic filariasis) and malaria among a migrant labour settlement in kerala, india. | Yes | Not applicable |
| 144 | DOI: 10.1089/env.2019.003 | Prevention of chronic diseases in climate change scenario in india | Yes | Not applicable |
| 145 | 10.1093/inthealth/ihaa056 | Progress and impact of 20 years of a lymphatic filariasis elimination programme in South-East Asia | Yes | Not applicable |
| 146 | 10.1186/s12936-016-1603-0 | Reduction in malaria prevalence and increase in malaria awareness in endemic districts of Bangladesh | Yes | Not applicable |
| 147 | 10.1186/1471-2334-14-440 | Risk factors for diarrhea hospitalization in Bangladesh, 2000-2008: a case-case study of cholera and shigellosis | Yes | Not applicable |
| 148 | PMID: 20562816 | Role of community empowerment in the elimination of lymphatic filariasis in south India | Yes | Not applicable |
| 149 | 10.4103/ijhas.IJHAS_35_16 | Seasonality of leptospirosis and its association with rainfall and humidity in Ratnagiri, Maharashtra | Yes | Not applicable |
| 150 | 10.4103/ijmr.IJMR_1638_15 | Seroprevalence of Japanese encephalitis virus & West Nile virus in Alappuzha district, Kerala | Yes | Not applicable |
| 151 | 10.3390/ijerph16183474 | Shift in Potential Malaria Transmission Areas in India, Using the Fuzzy-Based Climate Suitability Malaria Transmission (FCSMT) Model under Changing Climatic Conditions | Yes | Not applicable |
| 152 | 10.1371/journal.pntd.0003035 | Spatio-temporal distribution of dengue and lymphatic filariasis vectors along an altitudinal transect in Central Nepal | Yes | Not applicable |
| 153 | 10.1186/s13071-014-0540-4 | Species composition, seasonal occurrence, habitat preference and altitudinal distribution of malaria and other disease vectors in eastern Nepal. | Yes | Not applicable |
| 154 | 10.1093/infdis/jiab436 | Spread and Endemicity of Cholera in India: Factors Beyond the Numbers | Yes | Not applicable |
| 155 | 10.1007/s10666-017-9547-5 | Statistical Modeling of Health Effects on Climate-Sensitive Variables and Assessment of Environmental Burden of Diseases Attributable to Climate Change in Nepal | Yes | Not applicable |
| 156 | 10.14715/cmb/2018.64.4.8 | Status of lymphatic filariasis with progression of age and gender & eradication strategies: A survey among residents of Hardoi district of Uttar Pradesh, an endemic region of North India. | Yes | Not applicable |
| 157 | 10.1093/trstmh/trw067 | Surveillance for transmission of lymphatic filariasis in Colombo and Gampaha districts of Sri Lanka following mass drug administration | Yes | Not applicable |
| 158 | <https://doi.org/10.1093/trstmh/trt011> | Surveillance of lymphatic filariasis after stopping ten years of mass drug administration in rural communities in south India | Yes | Not applicable |
| 159 | 10.3390/ijgi7070275 | Temporal Variations and Associated Remotely Sensed Environmental Variables of Dengue Fever in Chitwan District, Nepal | Yes | Not applicable |
| 160 | 10.1371/journal.pone.0199579 | The association between temperature, rainfall and humidity with common climate-sensitive infectious diseases in Bangladesh | Yes | Not applicable |
| 161 | 10.1016/S0169-4758(00)01643-4 | The economic burden of lymphatic filariasis in india | Yes | Not applicable |
| 162 | doi.org/10.1016/S0001-706X(02)00030-X | The economic loss due to treatment costs and work loss to individuals with chronic lymphatic filariasis in rural communities of Orissa, India | Yes | Not applicable |
| 163 | 10.3402/gha.v8.29359 | The interrelationship between dengue incidence and diurnal ranges of temperature and humidity in a Sri Lankan city and its potential applications | Yes | Not applicable |
| 164 | 10.1016/s0277-9536(01)00230-1 | The spatial epidemiology of cholera in an endemic area of Bangladesh | Yes | Not applicable |
| 165 | 10.1007/s11136-013-0455-0 | The use of focus groups to develop a culturally relevant quality of life tool for lymphatic filariasis in Bangladesh. | Yes | Not applicable |
| 166 | 10.3329/jhpn.v31i1.14744 | Time series analysis of cholera in Matlab, Bangladesh, during 1988-2001 | Yes | Not applicable |
| 167 | 10.3389/fcimb.2021.641632 | Towards a Sustainable Vector-Control Strategy in the Post Kala-Azar Elimination Era | Yes | Not applicable |
| 168 | 10.1186/1475-2875-9-125 | Towards malaria risk prediction in Afghanistan using remote sensing | Yes | Not applicable |
| 169 | 10.3390/su9040604 | Towards sustainable public health surveillance in india: using routinely collected electronic emergency medical service data for early warning of infectious diseases | Yes | Not applicable |
| 170 | 10.1007/s00484-021-02097-x | Understanding the effect of climate change in the distribution and intensity of malaria transmission over India using a dynamical malaria model | Yes | Not applicable |
| 171 | 10.1111/nyas.13084 | Urban climate versus global climate change-what makes the difference for dengue? | Yes | Not applicable |
| 172 | 10.3402/gha.v9.30834 | Using health and demographic surveillance for the early detection of cholera outbreaks: analysis of community- and hospital-based data from Matlab, Bangladesh | Yes | Not applicable |
| 173 | PMID: 26418647 | Vector competence of two Indian populations of Culex quinquefasciatus (Diptera: Culicidae) mosquitoes to three West Nile virus strains | Yes | Not applicable |
| 174 | PMID: 22885260 | Vector control interventions for visceral leishmaniasis elimination initiative in South Asia, 2005-2010 | Yes | Not applicable |
| 175 | 10.3389/fpubh.2018.00238 | Vibrio cholerae Transmits Through Water Among the Household Contacts of Cholera Patients in Cholera Endemic Coastal Villages of Bangladesh, 2015-2016 (CHoBI7 Trial) | Yes | Not applicable |
| 176 | 10.1186/s41043-016-0040-6 | What is cholera? A preliminary study on caretakers' knowledge in Bangladesh | Yes | Not applicable |
| 177 | 10.1007/s00484-022-02404-0 | Time series analysis of leishmaniasis incidence in Sri Lanka: evidence for humidity-associated fluctuations. | Yes | Not applicable |
| 178 | 10.1016/j.amsu.2022.104936 | Cholera amid COVID-19: Call from three nations; India, Bangladesh, and Nepal | Yes | Not applicable |
| 179 | 10.1371/journal.pntd.0011820 | Economic burden of dengue in urban Bangladesh: A societal perspective. | Yes | Not applicable |
| 180 | <https://doi.org/10.1371/journal.pone.0292723> | Burden of dengue, leishmaniasis and lymphatic filariasis in India and its states from 1990–2019: Analysis from the Global Burden of Disease study (GBD 2019) | Yes | Not applicable |
| 181 | <https://doi.org/10.1016/j.eswa.2023.121490> | Modeling climate change impacts on vector-borne disease using machine learning models: Case study of Visceral leishmaniasis (Kala-azar) from Indian state of Bihar | Yes | Not applicable |
| 182 | <https://doi.org/10.1016/j.knosys.2023.110645> | Semantic web-based diagnosis and treatment of vector-borne diseases using SWRL rules | Yes | Not applicable |
| 183 | 10.1002/hsr2.1775 | Infectious diseases in Afghanistan: Strategies for health system improvement | Yes | Not applicable |
| 184 | 10.1371/journal.pone.0275447 | SARIMA and ARDL models for predicting leptospirosis in Anuradhapura district Sri Lanka. | Yes | Not applicable |
| 185 |  | The neglected role of relative humidity in the interannual variability of urban malaria in Indian cities | Yes | Not applicable |
| 186 | 10.4103/0972-9062.355958 | Vector and rodent surveillance for Orientia tsutsugamushi in north India. | Yes | Not applicable |
| 187 | 10.3855/jidc.15078 | infections in Pakistan: prevalence, factors affecting spread, and recommendations for controlZehra | Yes | Not applicable |
| **Information on studies excluded in the final literature screening process** | | | | |
| ID | **Link containing studies excluded in the study** | **Search terms used in database search** | **Date of Search** | **Reason for exclusion** |
| 1 | [https://pubmed.ncbi.nlm.nih.gov/rss/search/1n7mwSuZ06KmdojZz-acii1iPo30vrHex4R4215zJ62fDRXgVb/?limit=15&utm_campaign=pubmed-2&fc=20240816063006](https://pubmed.ncbi.nlm.nih.gov/?term=(Global%20warming)%20OR%20(climate%20change)%20OR%20(climate%20warming)%20OR%20(global%20heating)%20OR%20(greenhouse%20effect)%20AND%20(Communicable%20disease)%20OR%20(infectious%20disease)%20OR%20(transmittable%20disease*)%20OR%20(vector-borne%20disease)%20OR%20(water-borne%20disease)%20OR%20(food-borne%20disease)%20AND%20(forecast*)%20OR%20(early%20warning*)%20OR%20(risk%20assessment)%20OR%20(informatics)%20OR%20(risk%20mapping)%20OR%20(decision%20support)%20OR%20(preparedness)%20AND%20(South%20Asia)%20OR%20(India)%20OR%20(Bangladesh)%20OR%20(Sri%20Lanka)%20OR%20(Nepal)%20OR%20(Afghanistan)%20OR%20(Pakistan)%20OR%20(location)%20OR%20(Bhutan)) | (Global warming) OR (climate change) OR (climate warming) OR (global heating) OR (greenhouse effect) AND (Communicable disease) OR (infectious disease) OR (transmittable disease*) OR (vector-borne disease) OR (water-borne disease) OR (food-borne disease) AND (forecast*) OR (early warning*) OR (risk assessment) OR (informatics) OR (risk mapping) OR (decision support) OR (preparedness) AND (South Asia) OR (India) OR (Bangladesh) OR (Sri Lanka) OR (Nepal) OR (Afghanistan) OR (Pakistan) OR (location) OR (Bhutan) | 2/01/2022 | Inaccessible papers (n=13)  Records excluded due to not focusing on the subject matter (i.e., Climate and water sensitive diseases and hydrological/climate systems (n=588)  Reports excluded due to not focusing on the reviewed countries/ region (Bangladesh, Nepal, Sri Lanka, Bhutan, Pakistan, Afghanistan, and South Asia (n=74)  Records removed as duplicates by sci-wheel reference manger (n=229) |
| 2 | https://www.webofscience.com/wos/woscc/summary/43eaacdf-b010-4d59-9cbd-cb4880a5c6aa-0102374545/relevance/1 | (Global warming) OR (climate change) OR (climate warming) OR (global heating) OR (greenhouse effect) AND (relationship) OR (association) OR (connection) AND (meteorology) OR (temperature) OR (humidity) OR (precipitation) OR (rainfall) OR (hydrology) AND (Communicable disease) OR (infectious disease) OR (transmittable disease) OR (vector-borne disease) OR (water-borne disease) OR (food-borne disease)  AND (forecast) OR (early warning) OR (risk assessment) OR (informatics) OR (risk mapping) OR (decision support systems) OR (preparedness) AND (Bhutan, Pakistan, India, Sri Lanka, Nepal, Bangladesh, South Asia) | 30/04/2022 |  |
| 3 | <https://www.webofscience.com/wos/woscc/summary/ac01a1ee-59ce-4118-> ba6b-0f573d385b8e-c2228be9/relevance/1 | "Global warming" OR "climate change" OR "climate warming" OR "global heating" OR "greenhouse effect" AND "relationship" OR "association" OR "connection" AND "forecast" OR "early warning" OR "risk assessment" OR "informatics" OR "risk mapping" OR "decision support systems” “India" OR "Bangladesh" OR "Nepal" OR "Sri-Lanka" OR "Bhutan" OR "Pakistan" OR "Afghanistan" OR "south Asia" | 23/01/2024 |  |
| 4 | https://www.webofscience.com/wos/woscc/summary/7dbe4748-a59e-46dd-9d23-47125783efa6-c80fbce5/relevance/1 | (Global warming) OR (climate change) OR (climate warming) OR (global heating) OR (greenhouse effect) AND (Communicable disease) OR (infectious disease) OR (transmittable disease*) OR (vector-borne disease) OR (water-borne disease) OR (food-borne disease) AND (forecast*) OR (early warning*) OR (risk assessment) OR (informatics) OR (risk mapping) OR (decision support) OR (preparedness) AND (South Asia) OR (India) OR (Bangladesh) OR (Sri Lanka) OR (Nepal) OR (Afghanistan) OR (Pakistan) OR (location) OR (Bhutan) | 1/02/2024 |  |
