# Supplementary material for: Informing climate-health adaptation options through mapping the needs and potential for integrated climate-driven early warning forecasting systems in South Asia—A scoping review
Source: PLoS One. 2024 Oct 24;19(10):e0309757. doi: 10.1371/journal.pone.0309757 (PMC11500899; doi:10.1371/journal.pone.0309757)
Supplement: S1 File — (DOCX) [file pone.0309757.s009.docx]

**Table S1. Exemplar search strings used in Web of Science, PubMed and Scopus databases**

| Component | Search terms in Web of Science | Search terms in PubMed | Search terms in Scopus |
| --- | --- | --- | --- |
| Climate change | "Global warming" OR "climate change" OR "climate warming" OR "global heating" OR "greenhouse effect" AND "relationship" OR "association" OR "connection" | (Global warming) OR (climate change) OR (climate warming) OR (global heating) OR (greenhouse effect) | (Global warming) OR (climate change) OR (climate warming) OR (global heating) OR (greenhouse effect) AND (relationship) OR (association) OR (connection) AND (meteorology) OR (temperature) OR (humidity) OR (precipitation) OR (rainfall) OR (hydrology) |
| AND | AND | AND | AND |
| Climate-and water sensitive diseases | "Communicable disease*" OR "infectious disease" OR "transmittable disease*" OR "vector-borne disease" OR "water-borne disease" OR "food-borne disease" | (Communicable disease) OR (infectious disease) OR (transmittable disease*) OR (vector-borne disease) OR (water-borne disease) OR (food-borne disease) | (Communicable disease) OR (infectious disease) OR (transmittable disease) OR (vector-borne disease) OR (water-borne disease) OR (food-borne disease) |
| AND | AND | AND | AND |
| Early warning forecasting systems | "forecast" OR "early warning" OR "risk assessment" OR "informatics" OR "risk mapping" OR "decision support systems" | (forecast*) OR (early warning*) OR (risk assessment) OR (informatics) OR (risk mapping) OR (decision support) OR (preparedness) | (forecast) OR (early warning) OR (risk assessment) OR (informatics) OR (risk mapping) OR (decision support systems) OR (preparedness)))) |
| AND | AND | AND | AND |
| Country/ region focus | "India" OR "Bangladesh" OR "Nepal" OR "Sri-Lanka" OR "Bhutan" OR "Pakistan" OR "Afghanistan" OR "south Asia" | (South Asia) OR (India) OR (Bangladesh) OR (Sri Lanka) OR (Nepal) OR (Afghanistan) OR (Pakistan) OR (location) OR (Bhutan) | (Bhutan, Pakistan, India, Sri Lanka, Nepal, Bangladesh, south Asia) |
