# Supplementary material for: Informing climate-health adaptation options through mapping the needs and potential for integrated climate-driven early warning forecasting systems in South Asia—A scoping review
Source: PLoS One. 2024 Oct 24;19(10):e0309757. doi: 10.1371/journal.pone.0309757 (PMC11500899; doi:10.1371/journal.pone.0309757)
Supplement: S1 Graphical abstract — (PPTX) [file pone.0309757.s012.pptx]

## Slide 1
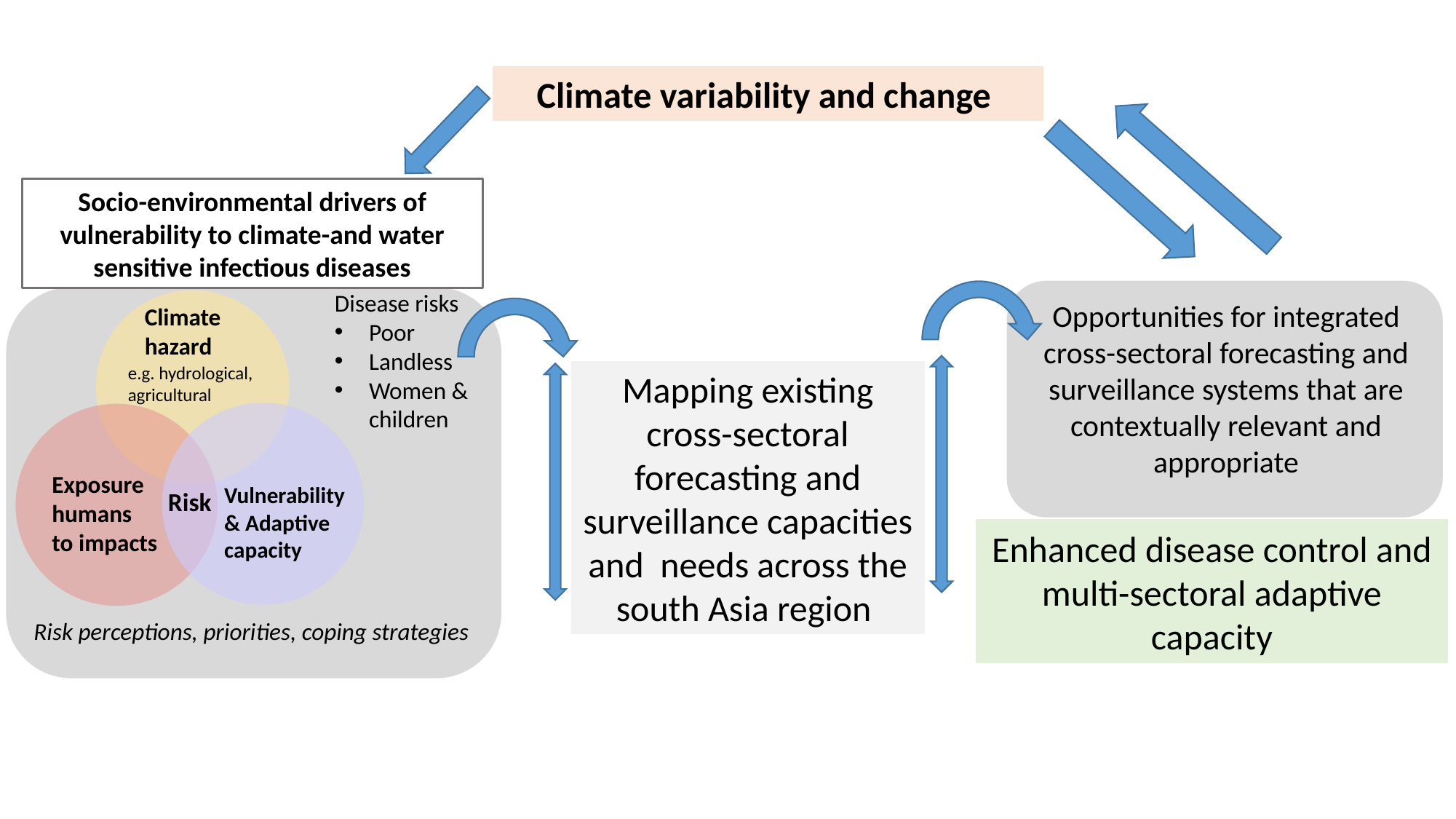

Climate variability and change
Socio-environmental drivers of vulnerability to climate-and water sensitive infectious diseases
Disease risks
Poor
Landless
Women & children
Opportunities for integrated cross-sectoral forecasting and surveillance systems that are contextually relevant and appropriate
Climate
hazard
e.g. hydrological,
agricultural
Mapping existing cross-sectoral forecasting and surveillance capacities and needs across the south Asia region
Exposure humans
to impacts
Vulnerability
& Adaptive capacity
Risk
Enhanced disease control and multi-sectoral adaptive capacity
Risk perceptions, priorities, coping strategies
