# Supplementary material for: Informing climate-health adaptation options through mapping the needs and potential for integrated climate-driven early warning forecasting systems in South Asia—A scoping review
Source: PLoS One. 2024 Oct 24;19(10):e0309757. doi: 10.1371/journal.pone.0309757 (PMC11500899; doi:10.1371/journal.pone.0309757)
Supplement: Attachment — Submitted filename: Response to editors comments.docx [file pone.0309757.s013.docx]

**Response to Editor’s comments on manuscript (PONE-D-23-38984- [EMID:2e59ded307b48990]) – ‘Informing climate-health adaptation options through mapping the needs and potential for integrated climate-driven early warning forecasting systems in South Asia’**

**Overview of changes made**

Thank you again to the Editor for the comments which we addressed below:

**Editor’s comments:**

1. Thank you for submitting your manuscript for review by PLOS ONE. After careful consideration, we have decided that your manuscript does not meet our standards for systematic and scoping reviews. We are therefore rejecting your manuscript.

PLOS ONE considers systematic reviews and meta-analyses for publication; however, these submissions must address a clear and defined research question; conduct a systematic and comprehensive literature review; and use clearly reported, reproducible, and systematic methods to identify, select, and critically appraise relevant research. Our author guidelines for systematic reviews are at <https://journals.plos.org/plosone/s/submission-guidelines#loc-systematic-reviews-and-meta-analyses>.

In the case of your submission, we are concerned that the literature review was completed in April 2022. Systematic and scoping reviews should provide an up-to-date overview of the research topic covered. Since the literature search was completed over a year ago, we are concerned that this study does not meet this requirement.

As a result of these concerns, we cannot consider the manuscript for publication at PLOS ONE at this time. However, if you are able to address the concerns discussed above, we may be able to consider a revised version of the manuscript. This would however require a substantial amount of work and thus, would be considered as a new submission. If you are able to revise the manuscript to respond to the concerns noted above, please refer to the original submission in the covering letter and accompany the submission by a rebuttal letter that responds to the comments made on the original manuscript.

*Response: thank you for the comment. We have revised the manuscript by updating the literature search as suggested. Please related text in the revised manuscript below (lines 169-172):*

*“Both the peer-reviewed literature and policy searches were conducted between 2nd January 2022 and 30th April 2022. A further peer-reviewed literature search was carried out (using the same search terms) between 23rd January 2024 and 1st February 2024 based on which the corpus of relevant literature was updated.”*

2. Please also ensure that you submit a PRISMA - ScR checklist, if you resubmit your manuscript upon revision.

*Response: We have appended a completed PRISMA-SCR checklist to the revised manuscript (see supplementary information 2)*

Thank you for the comments again.
